# Supplementary material for: Short-term effects of cold spells on hospitalisations for acute exacerbation of chronic obstructive pulmonary disease: a time-series study in Beijing, China
Source: BMJ Open. 2021 Jan 6;11(1):e039745. doi: 10.1136/bmjopen-2020-039745 (PMC7789453; doi:10.1136/bmjopen-2020-039745)
Supplement: Supplementary data [file bmjopen-2020-039745supp002.pdf]

**Table S2** The cumulative effects of cold spells under the optimal definition using different degrees of freedom for time per year in the DLM model

| df for time per year | Group | CRR (95% CI) | | | |
|---|---|---|---|---|---|
| | | Lag0 | Lag0-7 | Lag0-14 | Lag0-21 |
| 3[a] | Total | 1.042 (1.013-1.072)* | 1.249 (1.136-1.374)* | 1.343 (1.206-1.496)* | 1.394 (1.193-1.630)* |
| | Male | 1.042 (1.011-1.074)* | 1.243 (1.123-1.375)* | 1.316 (1.173-1.477)* | 1.342 (1.136-1.586)* |
| | Female | 1.041 (1.005-1.077)* | 1.257 (1.119-1.411)* | 1.383 (1.215-1.574)* | 1.476 (1.211-1.783)* |
| | Age<65 | 1.017 (0.972-1.064) | 1.120 (0.963-1.303) | 1.159 (0.977-1.376) | 1.107 (0.862-1.422) |
| | Age≥65 | 1.046 (1.017-1.077)* | 1.275 (1.158-1.404)* | 1.382 (1.240-1.540)* | 1.456 (1.244-1.705)* |
| 4 | Total | 1.023 (0.992-1.055) | 1.150 (1.033-1.280)* | 1.218 (1.078-1.376)* | 1.233 (1.036-1.467)* |
| | Male | 1.029 (0.997-1.063) | 1.173 (1.045-1.316)* | 1.226 (1.074-1.399)* | 1.225 (1.015-1.480)* |
| | Female | 1.011 (0.974-1.050) | 1.107 (0.971-1.262) | 1.196 (1.034-1.385)* | 1.232 (0.998-1.520) |
| | Age<65 | 1.010 (0.961-1.061) | 1.077 (0.906-1.280) | 1.105 (0.907-1.345) | 1.056 (0.795-1.401) |
| | Age≥65 | 1.026 (0.995-1.058) | 1.166 (1.046-1.300)* | 1.244 (1.100-1.406)* | 1.272 (1.067-1.517)* |
| 5 | Total | 1.022 (0.990-1.056) | 1.144 (1.008-1.298)* | 1.208 (1.031-1.415)* | 1.222 (0.978-1.526) |
| | Male | 1.030 (0.994-1.066) | 1.171 (1.021-1.343)* | 1.222 (1.027-1.452)* | 1.223 (0.960-1.559) |
| | Female | 1.009 (0.970-1.050) | 1.093 (0.938-1.275) | 1.174 (0.972-1.418) | 1.203 (0.923-1.569) |
| | Age<65 | 1.011 (0.959-1.066) | 1.082 (0.883-1.327) | 1.127 (0.874-1.453) | 1.127 (0.788-1.612) |
| | Age≥65 | 1.025 (0.992-1.059) | 1.158 (1.019-1.316)* | 1.227 (1.045-1.440)* | 1.245 (0.994-1.559) |

CI, confidence interval; df, degree of freedom; RR, relative risk.

*$P$<0.05.

[a]Used in the study.
