# Supplementary material for: Short-term effects of cold spells on hospitalisations for acute exacerbation of chronic obstructive pulmonary disease: a time-series study in Beijing, China
Source: BMJ Open. 2021 Jan 6;11(1):e039745. doi: 10.1136/bmjopen-2020-039745 (PMC7789453; doi:10.1136/bmjopen-2020-039745)
Supplement: Supplementary data [file bmjopen-2020-039745supp003.pdf]

Table S3 The cumulative effects of cold spells under the optimal definition using different degrees of freedom for relative humidity in the DLM model

df for relative humidity	Group	CRR (95% CI)			
		Lag0	Lag0-7	Lag0-14	Lag0-21
3[a]	Total	1.042 (1.013-1.072)*	1.249 (1.136-1.374)*	1.343 (1.206-1.496)*	1.394 (1.193-1.630)*
	Male	1.042 (1.011-1.074)*	1.243 (1.123-1.375)*	1.316 (1.173-1.477)*	1.342 (1.136-1.586)*
	Female	1.041 (1.005-1.077)*	1.257 (1.119-1.411)*	1.383 (1.215-1.574)*	1.476 (1.211-1.783)*
	Age<65	1.017 (0.972-1.064)	1.120 (0.963-1.303)	1.159 (0.977-1.376)	1.107 (0.862-1.422)
	Age≥65	1.046 (1.017-1.077)*	1.275 (1.158-1.404)*	1.382 (1.240-1.540)*	1.456 (1.244-1.705)*
4	Total	1.041 (1.012-1.071)*	1.248 (1.135-1.373)*	1.343 (1.205-1.495)*	1.392 (1.190-1.629)*
	Male	1.042 (1.011-1.073)*	1.240 (1.120-1.372)*	1.314 (1.170-1.475)*	1.336 (1.130-1.580)*
	Female	1.041 (1.006-1.078)*	1.259 (1.120-1.414)*	1.385 (1.217-1.578)*	1.482 (1.225-1.792)*
	Age<65	1.017 (0.972-1.064)	1.119 (0.961-1.302)	1.158 (0.975-1.375)	1.105 (0.859-1.420)
	Age≥65	1.046 (1.017-1.076)*	1.274 (1.157-1.403)*	1.381 (1.239-1.540)*	1.455 (1.242-1.704)*
5	Total	1.041 (1.012-1.071)*	1.244 (1.131-1.369)*	1.337 (1.200-1.490)*	1.383 (1.182-1.619)*
	Male	1.041 (1.010-1.073)*	1.236 (1.116-1.368)*	1.307 (1.164-1.468)*	1.327 (1.121-1.569)*
	Female	1.040 (1.005-1.077)*	1.256 (1.117-1.411)*	1.381 (1.212-1.574)*	1.474 (1.218-1.785)*
	Age<65	1.015 (0.970-1.062)	1.111 (0.955-1.293)	1.148 (0.967-1.363)	1.089 (0.847-1.400)
	Age≥65	1.046 (1.016-1.076)*	1.271 (1.154-1.400)*	1.377 (1.234-1.536)*	1.448 (1.235-1.697)*

CI, confidence interval; df, degree of freedom; RR, relative risk.

*P<0.05.

[a]Used in the study.
