# Supplementary material for: Short-term effects of cold spells on hospitalisations for acute exacerbation of chronic obstructive pulmonary disease: a time-series study in Beijing, China
Source: BMJ Open. 2021 Jan 6;11(1):e039745. doi: 10.1136/bmjopen-2020-039745 (PMC7789453; doi:10.1136/bmjopen-2020-039745)
Supplement: Author's manuscript [file bmjopen-2020-039745.draft_revisions.pdf]

BMJ Open

BMJ Open is committed to open peer review. As part of this commitment we make the peer review history of every article we publish publicly available.

When an article is published we post the peer reviewers' comments and the authors' responses online. We also post the versions of the paper that were used during peer review. These are the versions that the peer review comments apply to.

The versions of the paper that follow are the versions that were submitted during the peer review process. They are not the versions of record or the final published versions. They should not be cited or distributed as the published version of this manuscript.

BMJ Open is an open access journal and the full, final, typeset and author-corrected version of record of the manuscript is available on our site with no access controls, subscription charges or pay-per-view fees (http://bmjopen.bmj.com).

If you have any questions on BMJ Open's open peer review process please email
info.bmjopen@bmj.com

BMJ Open

The short-term effects of cold spells on hospitalizations for chronic obstructive pulmonary disease: a time-series study in Beijing, China

Journal:	*BMJ Open*
Manuscript ID	bmjopen-2020-039745
Article Type:	Original research
Date Submitted by the Author:	24-Apr-2020

Complete List of Authors:	Liu, Yanbo; Peking Union Medical College Hospital, Internal Medicine<br>Chen, Yuxiong; Peking Union Medical College Hospital<br>Kong, Dehui; Peking Union Medical College Hospital<br>Liu, Xiaole; Peking Union Medical College Hospital<br>Fu, Jia; Peking Union Medical College Hospital<br>Zhang, Yongqiao; Peking Union Medical College Hospital<br>Zhao, Yakun; Peking Union Medical College Hospital<br>Chang, Zhen'ge; Peking Union Medical College Hospital<br>Zhao, Xiaoyi; Peking Union Medical College Hospital<br>Xu, Kaifeng; Peking Union Medical College Hospital<br>Jiang, Chengyu; Chinese Academy of Medical Sciences and Peking Union Medical College State Key Laboratory of Medical Molecular Biology<br>Fan, Zhongjie; Peking Union Medical College Hospital

Keywords:	Epidemiology < TROPICAL MEDICINE, PUBLIC HEALTH, Chronic airways disease < THORACIC MEDICINE

SCHOLARONE™
Manuscripts

I, the Submitting Author has the right to grant and does grant on behalf of all authors of the Work (as defined in the below author licence), an exclusive licence and/or a non-exclusive licence for contributions from authors who are: i) UK Crown employees; ii) where BMJ has agreed a CC-BY licence shall apply, and/or iii) in accordance with the terms applicable for US Federal Government officers or employees acting as part of their official duties; on a worldwide, perpetual, irrevocable, royalty-free basis to BMJ Publishing Group Ltd ("BMJ") its licensees and where the relevant Journal is co-owned by BMJ to the co-owners of the Journal, to publish the Work in this journal and any other BMJ products and to exploit all rights, as set out in our licence.

The Submitting Author accepts and understands that any supply made under these terms is made by BMJ to the Submitting Author unless you are acting as an employee on behalf of your employer or a postgraduate student of an affiliated institution which is paying any applicable article publishing charge ("APC") for Open Access articles. Where the Submitting Author wishes to make the Work available on an Open Access basis (and intends to pay the relevant APC), the terms of reuse of such Open Access shall be governed by a Creative Commons licence – details of these licences and which Creative Commons licence will apply to this Work are set out in our licence referred to above.

Other than as permitted in any relevant BMJ Author's Self Archiving Policies, I confirm this Work has not been accepted for publication elsewhere, is not being considered for publication elsewhere and does not duplicate material already published. I confirm all authors consent to publication of this Work and authorise the granting of this licence.

For peer review only

The short-term effects of cold spells on hospitalizations for chronic obstructive pulmonary disease: a time-series study in Beijing, China

Authors: Yanbo Liu[1#], Yuxiong Chen[1#], Dehui Kong[1], Xiaole Liu[1], Jia Fu[1], Yongqiao Zhang[1], Yakun Zhao[1], Zhen'ge Chang[1], Xiaoyi Zhao[2], Kaifeng Xu[1], Chengyu Jiang[3], Zhongjie Fan[1*]

Affiliation and Address:

[1]Department of Medicine, Peking Union Medical College Hospital, Peking Union Medical College & Chinese Academy of Medical Sciences, No.1 Shuaifuyuan Wangfujing Dongcheng District, Beijing 100730, China.

[2]Department of Physiotherapy, Peking Union Medical College Hospital, Peking Union Medical College & Chinese Academy of Medical Sciences, No.1 Shuaifuyuan Wangfujing Dongcheng District, Beijing 100730, China.

[3]The State Key Laboratory of Medical Molecular Biology, Institute of Basic Medical Sciences, Chinese Academy of Medical Sciences, Department of Biochemistry, Peking Union Medical College, Beijing 100005, China.

[#]These authors contributed equally to this work.

[*]**Corresponding author:**

Zhongjie Fan, Department of Medicine, Peking Union Medical College Hospital, Peking Union Medical College & Chinese Academy of Medical Sciences, No.1 Shuaifuyuan Wangfujing Dongcheng District, Beijing 100730, China (0086-13901199579, Fanzhongjie@pumch.cn)

Keywords: cold spells, chronic obstructive pulmonary disease, hospitalizations, intensity and duration

For peer review only

For peer review only - http://bmjopen.bmj.com/site/about/guidelines.xhtml

ABSTRACT

Objectives: Our work aimed at exploring the relationship between cold spells and chronic obstructive pulmonary diseases (COPD) hospitalizations in Beijing, China, and assessing the moderating effects of the intensities and the durations of cold spells, as well as identifying the vulnerable.

Design: Time-series study.

Setting: We obtained time-series data of COPD hospitalizations, meteorological variables and air quality index in Beijing, China during 2012–2016.

Participants: All COPD hospitalizations among permanent residents in Beijing, China in cold seasons (November to March) during 2012-2016 were included. (n=84,571).

Primary and secondary outcome measures: A quasi-Poisson regression with a distributed lag model was fitted to investigate the short-term effects of cold spells on COPD hospitalizations by comparing the counts of COPD admissions during cold spell days with those during non-cold spell days.

Results: Cold spells under different definitions were associated with increased risks of COPD hospitalizations, with the maximum cumulative relative risk (CRR) over three weeks (lag0-21). The cumulative effects at lag0-21 increased with the intensities and the durations of cold spells. Under the optimal definition, the most significant single-day relative risk (RR) was found on the days of cold spells (lag0) with RR=1.042 (95%CI: 1.013, 1.072), and the CRR at lag0-21 was 1.394 (95%CI: 1.193, 1.630). The elderly (aged≥65) were more vulnerable to the effects of cold spells on COPD hospitalizations.

Conclusion: Cold spells are associated with increased COPD hospitalizations in Beijing, with the cumulative effects increased with their intensities and durations. The elderly are at particular risk of COPD hospitalizations triggered by cold spells.

For peer review only - http://bmjopen.bmj.com/site/about/guidelines.xhtml

Strengths and limitations of this study:

- This study was the first to examine the association between cold spells and COPD hospitalizations in China.
- The study assessed the effects of cold spells under different definitions on COPD hospitalizations, thereby finding out a more reasonable cold spell definition on the issue.
- The study identified more vulnerable subpopulation to COPD hospitalizations in response to cold spells, which guide more targeted prevention strategies.
- The ecological design cannot imply causality definitely, while limited information on individual-level factors and inevitable exposure measurement errors may lead to bias.
- The data from only one city limited the generalization of the study findings.

INTRODUCTION

The Intergovernmental Panel on Climate Change (IPCC) has predicted that human activities and global climate change cause variations in frequency, intensity and duration of many extreme weather events, including heatwaves and cold spells.[1] Although the amount of cold spells may decrease over most land areas due to global warming, a few recent studies found that the persistent shift of the Arctic polar vortex and Arctic amplification associated with global warming could lead to increased extremely cold events in mid-latitudes.[2, 3] Over the last few years, the impacts of cold spells on human health have gained growing attention from the public. Many studies have reported positive relationships between cold spells and mortality[4-6] while the impacts of cold spells on hospital visits or admissions are under-examined.

For peer review only

Chronic obstructive pulmonary disease (COPD) is one of the common respiratory diseases characterized by poorly reversible limitation of airflow.[7] Owing to its high prevalence, morbidity, mortality and economic burden globally, COPD has been an important public health concern and will remain a huge challenge for healthcare practitioners in the foreseeable future.[8] Thus, it is crucial to identify the risk factors of COPD, to improve strategies on prevention and intervention. Given the projected climate change, extreme temperature events potentially pose threats to COPD patients. Many epidemiological studies have indicated that COPD has higher rates of exacerbation and hospitalization in cold seasons.[9-11] We hypothesized that cold spells, defined as prolonged periods of extremely cold weather, may be more relevant to COPD hospitalizations. However, few studies have been carried out on the very issue.[12]

As the world’s' largest country by population, China shoulders the enormous burden of COPD. A national cross-sectional study from 2012 to 2015 showed that the prevalence of COPD among Chinese adults aged 20 years and older was 8.6% (99.9 million patients).[13] On the other hand, with most areas located in mid-latitudes, China has experienced several severe cold spells in recent years. The cold spells in 2008 resulted in a significantly higher all-cause mortality in subtropical China and estimated losses exceeding $22.3 billion.[14, 15] Moreover, the public now has a better perception of the potential risks of extreme temperatures in China, especially those with chronic conditions.[16] So it is of great value to assess the association between cold spells and COPD hospitalizations to build prevention and adaption strategies in China.

Cold spells have been defined differently due to the heterogeneity of climate and people's adaptive capacities in different regions. Previous studies suggested that the effects of cold spells varied by different cold spell characteristics and individual-specific factors.[17, 18] We have three main objectives in this work: (1) to illuminate the short-

For peer review only

For peer review only - http://bmjopen.bmj.com/site/about/guidelines.xhtml

term effects of cold spells on the risk of hospitalizations for COPD with time-series methods. (2) to investigate the modification effect of cold spell intensities and durations by fitting different definitions. (3) to identify potentially vulnerable populations through stratified analyses. The results could help better understand the relationship between extremely cold events and COPD hospitalizations, and provide scientific evidence in policymaking for the prevention and the intervention of COPD.

MATERIALS AND METHODS

Data collection

Beijing, the capital of China, is located in the northern part of China (39°56′N, 116°20′E). The area covers 16410.54 km^2, with more than 21 million population in 2016. Beijing has a typical semi-humid continental monsoon climate with four distinctive seasons.

Daily hospitalizations for COPD from January 1, 2012, to December 31, 2016, were collected from Beijing Municipal Health Commission Information Center covering all the secondary and tertiary hospitals in Beijing. All case files consisted of the following information: admission date, discharge date, age, gender, address, diagnosis and International Classification of Diseases 10th revision (ICD-10) diagnostic code. Those among Beijing residents with COPD as the primary discharge diagnosis (coded as J44) were included in the study. The study was approved by the ethical committee of Peking Union Medical College Hospital. All the data for the analysis were anonymous at collection.

We collected the daily 2012–2016 meteorological data in Beijing from the China Meteorological Data Sharing Service System, including daily mean temperature (℃), daily mean relative humidity (%) and daily mean air pressure (hPa). For the same period,

For peer review only - http://bmjopen.bmj.com/site/about/guidelines.xhtml

the daily air quality index (AQI) was obtained from the China National Environmental Monitoring Centre. The AQI value denotes the maximum value of individual air quality indexes (IAQI) of six monitored air pollutants (particulate matter with aerodynamic diameter<2.5μm, particulate matter with aerodynamic diameter<10μm, nitrogen dioxide, sulfur dioxide, carbon monoxide, and ozone). Considering the impact of influenza on COPD exacerbations,[19] we also accessed the data on influenza isolates from the Chinese National Influenza Center. Influenza epidemics (a binary variable representing days with relatively high influenza incidence) were defined when the proportion of isolates positive for influenza exceeded 30% of the maximum seasonal level (Influenza surveillance season was defined from the 27th week of the previous year to the 26th week of the following year).[20]

For peer review only

Cold spell definitions

The definition of cold spells varied across the research field due to distinct climatic features and temperature variations in different regions. As to the prior studies, cold spells were usually defined based on their temperature thresholds and durations.[4, 12, 21] Instead of specific temperatures as thresholds, percentiles of temperature were shown to have a better model fit according to quasi-Poisson Akaike Information Criterion (Q-AIC).[4] Moreover, some researchers suggested that daily mean temperature is superior to the minimum or maximum temperature as an indicator to define cold spells because it reflects the exposure throughout the day rather than a short period.[17, 22] Therefore, we defined cold spell episodes as days when daily mean temperature at or below the 10th, 5th or 3rd percentile for at least 2, 3 or 4 consecutive days of the study period.[18] To avoid the possible biases caused by a few extreme summer events, we restricted the study period to the five coldest adjacent months (from November of the

For peer review only - http://bmjopen.bmj.com/site/about/guidelines.xhtml

previous year to March of the following year) for each year.[6, 12, 17] The cold spell was treated as a dichotomous variable, with a value of 1 during the cold spell period. The statistical analyses were performed separately for each definition of cold spells.

Statistical methods

In the analyses, the dependent variable was the number of daily COPD hospitalizations following a quasi-Poisson distribution. Hence, we adopted a distributed lag model (DLM) [23] with a quasi-Poisson generalized linear regression model. To investigate the effects of cold spells on COPD hospitalizations, we compared the counts of COPD admissions during cold spell days with those during non-cold spell days, after adjusting for relative humidity, atmospheric pressure, AQI, seasonality, long-term trends, statutory holiday, influenza epidemics, and day of the week (DOW). The Q-AIC was employed to choose the optimal cold spell definition and degrees of freedom (df). The model was established as follows:

$$Y_t \sim \mathrm{quasiPoisson}(\mu_t)$$

$$\mathrm{Log}(\mu_t) = \alpha + \mathrm{cb}(\mathrm{CS}_t, \mathrm{lag}, \mathrm{df}=3) + \mathrm{ns}(\mathrm{RH}_t, \mathrm{df}=3) + \mathrm{ns}(\mathrm{AP}_t, \mathrm{df}=3) + \mathrm{ns}(\mathrm{AQI}_t, \mathrm{df}=3) + \mathrm{ns}(\mathrm{Time}_t, \mathrm{df} = {}^{3}/\mathrm{per\ year}) + \gamma \mathrm{DOW}_t + \delta \mathrm{Holiday}_t + \nu \mathrm{Influenza}_t ,$$

where t is the day of observation; Y_t is the expected number of hospitalizations for COPD on day t; α is the intercept; CS_t denotes the cold spells on day t (0=non-cold spell days, and 1=cold spell days); cb represents the cross-basis function, including a linear function and a natural cubic spline function with 3 df to assess the linear and lagged effects of the cold spells separately. We fitted a lag structure up to 21 days in the models to completely capture the flexible lagged effects of cold spells exposure. ns refers to the natural cubic spline function. ns with 3 df was applied for the mean relative humidity (RH_t), mean atmospheric pressure (AP_t) and air quality index (AQI_t), respectively. ns with 3 df per year

For peer review only - http://bmjopen.bmj.com/site/about/guidelines.xhtml

was used to control the seasonality and long-term trends. DOW_t is a categorical variable indicating the day of the week on day t, and γ is the coefficient. $Holiday_t$ is a binary variable (0=non-statutory holiday, and 1=statutory holiday) and δ is the coefficient. $Influenza_t$ is a dichotomous variable with the value of "1" for the influenza epidemic on day t, and ν is the coefficient. The statistical methods, maximum lag days and confounding factors included in the model were commonly utilized and described in previous publications.[4, 14, 17, 18, 24, 25]

To observe the variation trend of lagged effects, we calculated the single-day lagged effects (from lag0 to lag21) and cumulative effects (lag0, lag0–7, lag0–14 and lag0–21) of cold spells on COPD hospitalizations, respectively. To identify the susceptible subpopulations for more targeted public health interventions, we further conducted subgroup analyses to investigate the potential modification effects by gender (male and female) and age (0-64 years old and ≥65 years old) under the optimal definition of cold spells. The statistical differences of the risk estimates between the subgroups were examined by the Z-test with the following equation:

$$Z = (E_1 - E_2)/\sqrt{(SE_1{}^2 + SE_2{}^2)}$$

where Z represents the Z-test value; E_1 and E_2 denote the effect estimates of two categories; SE_1 and SE_2 are corresponding standard errors of E_1 and E_2.

Sensitivity analysis

We performed the sensitivity analyses by altering the df with 3–5 df per year of the long-term trend, 3–5 df of the relative humidity, 3-5 df of the air pressure, 3-5 df of the AQI and 3–5 df of the lag dimension in the DLM under the optimal definition of cold spells. We used R software version 3.6.1 with the “dlnm” and “splines” packages to run the analyses. All statistical tests were two-sided and values of p<0.05 were considered

For peer review only

For peer review only - http://bmjopen.bmj.com/site/about/guidelines.xhtml

statistically significant.

Patient and public involvement

Patients were not involved in development of the research question and outcome measures, study design or conduct of this study.

RESULTS

Data description

Table 1 shows the descriptive statistics of the study population, meteorological variables and AQI during the cold seasons (November to March) from 2012 to 2016 in Beijing. There was a total of 84,571 COPD hospitalizations throughout the study period. Among these cases, 63.6% were males and 36.4% were females. 83.9% of all patients were aged 65 years and above. The average of daily mean temperature, relative humidity, air pressure, and AQI were 0.9℃ (range, -16.0-18.0℃), 46.6% (range, 8.0-98.0%), 1025.3hPa (range, 1005.0-1044.0hPa) and 126.3 (range, 17.0-485.0), respectively.

Table 2 shows the overview information of cold spells under different definitions. More days were defined as cold spell days with higher temperature thresholds and shorter duration. We observed the most cold spell episodes and days in four years with the definition of periods for at least 2 days and daily mean temperature below or at the 10th percentile (-6℃). In contrast, there were only 2 cold spell episodes and 10 cold spell days if we defined cold spells as periods for 4 or more consecutive days when daily mean temperature was below or at the 3rd percentile (-8℃).

Effects of cold spells under different definitions

For peer review only - http://bmjopen.bmj.com/site/about/guidelines.xhtml

Figure 1 depicts the lag structures of associations between cold spells under 9 different definitions and COPD hospitalizations of the total population. All cold spells had impacts on the risks of hospitalizations for COPD and most trends of their lagged effects were non-linear with two patterns. One was that the relative risk (RR) of hospitalizations for COPD reached maximum on the days (lag0) of cold spells, then decreased and remained significant for 10-16 days (lag10-lag16). The other one was that the RR became significant on the 3rd or 4th day (lag3 or lag4) after exposure to cold spells, then gradually reached the maximum at about the 8th day (lag8) and lasted till the 15th-17th days (lag15-lag17).

Table 3 shows the cumulative effects of cold spells on COPD hospitalizations under different definitions. For each definition, the cumulative relative risk (CRR) increased with longer cumulative lags, with the highest CRR at lag0-21. Among 9 different definitions, the CRRs of cold spells at lag0-21 increased as the definition had a longer duration or lower temperature threshold. The maximum CRR over lag0-21 when the temperature threshold was set at the 10th percentile appeared at the duration≥4 consecutive days. In comparison, the CRR values of the duration≥3 consecutive days and ≥2 consecutive days were lower. Likewise, when the duration was set for at least 4 consecutive days, the maximum CRR over lag0-21 appeared at the temperature range defined as $\leq 3^{rd}$ percentile, while the CRR of temperature threshold at the 5th and 10th percentile were lower. The lowest Q-AIC value (7769.8) indicating the best model fit was observed in Model 3 (Table 2). Hence, the optimal cold spell definition was daily mean temperature $\leq 10^{th}$ percentile (-6℃) with at least 4 consecutive days during the study period. The optimal model was able to find the most significant single-day lagged effect earliest (lag0) and remained significant till the 14th day (lag14) (Figure 1).

Table 4 and Figure 2 reveal the results for the subgroup analyses of gender and age

For peer review only

For peer review only - http://bmjopen.bmj.com/site/about/guidelines.xhtml

based on the optimal cold spell definition. The effects of cold spells were similar between males and females, with the most significant single-day lagged effect both occurred at lag0 (Z=0.041, P=0.48). The cumulative effects at lag0-21 of two genders also differed slightly (Z=-0.730, P=0.23). Additionally, in the subgroups stratified by the age, the most significant single-day lagged effect and cumulative effect for people aged≥65 years were at lag0 and at lag0-21, respectively. However, no significant effect of cold spells was observed in those aged 0-64 years. The results of sensitivity analyses indicated that the effect estimates of cold spells under the optimal definition on COPD hospitalizations were still robust. (See Supplementary Materials, Tables S1–S5).

DISCUSSION

In this study, we showed that cold spells were associated with increase hospitalizations for COPD. The adverse impacts of cold spells varied with their durations and intensities. Based on the statistical model fit, the optimal definition of cold spells was daily mean temperature less than or equal to the 10th percentile lasting for at least 4 consecutive days during the study period. The elderly seemed more sensitive to cold spells than the younger, while the susceptibility difference between genders was not noticeable.

Our finding is in accordance with previous studies reporting an excess of COPD hospitalizations,[12] emergency visits[26] and mortality[4, 6, 26] associated with cold spells. For instance, Monteiro et al. reported significant effects of cold spells identified by various indices on COPD hospitalizations with a lagged effect of at least two weeks.[12] Several underlying mechanisms may explain for elevated COPD morbidity and mortality attributable to extremely cold events. Firstly, cold exposure has been found to be related to a decline in lung function (FEV_1, FVC and PEF) among COPD patients.[27, 28] Secondly, Koskela et al. reported that cold air could directly induce bronchoconstriction,

For peer review only - http://bmjopen.bmj.com/site/about/guidelines.xhtml

leading to excessive dyspnea in patients with COPD.[29] Thirdly, cold exposure may suppress the immune response and increase susceptibility to viral infections in humans.[30] Meanwhile, the transmission efficiency of the influenza virus is inversely correlated with ambient temperature.[31] Fourthly, the cold temperature may provoke airway inflammation and mucin hypersecretion in airway epithelium, which results in COPD morbidity and mortality by blocking airways and causing recurrent infections.[32, 33]

We found that the CRR values of COPD hospitalizations increased with longer duration and higher intensity of cold spells. Some prior studies have similar findings,[17, 18] indicating that both the duration and the intensity affect the health risks of cold spells. According to the minimum Q-AIC value criterion, the optimal definition of cold spells was the daily average temperature at or below the 10th percentile with 4 or more consecutive days. Compared with other definitions, this one had a lower intensity and longer duration, and the most significant single-day lagged effect of cold spells on COPD hospitalizations appeared earliest (lag0). Our results agree with a study in Porto showing that the moderately low temperature with long periods contributed greater to COPD exacerbation than extremely low temperature with shorter-lasting days.[12] However, studies on some other diseases defined the optimal cold spell definitions with a threshold at the 5th percentile or at least 2 days duration,[18, 24] indicating different definitions may apply to different outcomes. In addition, some studies reported the temporal changes of people's adaption capacities to cold spells during recent decades under climate change.[5, 34] Overall, more effective cold spell definitions and warning systems adapted to regional climate, specific diseases, and dynamic changes of the population's sensitivity should be further studied and implemented in the future.

In the subgroup analyses based on the optimal cold spell definition, we found that the

For peer review only

For peer review only - http://bmjopen.bmj.com/site/about/guidelines.xhtml

effects of cold spells on COPD hospitalizations were more significant in the elderly (aged ≥65 years) than people aged 0–64 years. This finding is consistent with previous studies.[4, 35, 36] The reasons may point to reduced thermoregulatory ability, higher prevalence of chronic diseases[17, 26] and impaired immunity[37] in the elderly. Note that since aging is one of the risk factors of COPD, most patients in our study were over 64, giving more power to achieve statistical significance. However, some studies showed the opposite results.[6] It was speculated that the younger tend to spend more time outdoors, which increase opportunities for exposure to extremely cold temperature. In terms of gender, we found similar impacts of cold spells among males and females, which corresponds with previous studies.[4, 35, 36]

Our study showed substantial effects of cold spells on the risk of COPD hospitalizations with a lagged effect of about 3 weeks. As a result, it is meaningful and urgent to provide effective and practical guidelines for preventions, particularly for COPD patients in response to cold spells in China. Both the government and individuals should take practical actions. The meteorological departments should improve early warning systems with timely forecast and publication of extremely cold events. Moreover, the government should exert great efforts to raise public awareness of the health hazards of cold spells and ensure adequate public and medical services coping with cold spells.[4] As for individuals, it has been reported that staying indoor and wearing warm clothing could reduce mortality in extremely cold weather.[38] Tseng et al. suggested that COPD patients who received inhaled medicine were less affected by cold temperature-related COPD exacerbation.[36] Therefore, keeping warm, minimizing the outdoor activities and taking medication regularly are vital measures to fight against cold spells for individuals with COPD.

The main strengths of our study are as follows: Firstly, to the best of our knowledge,

For peer review only

For peer review only - http://bmjopen.bmj.com/site/about/guidelines.xhtml

this is the first study to investigate the relationship between cold spells and COPD hospitalizations in China. Secondly, we controlled the confounding factors of air pollution and influenza infections, which were not considered by some previous studies. Existing literature has shown that both air pollutants and influenza may influence the health effects of low temperatures.[38, 39] Thirdly, we identified the elderly more vulnerable to cold spells by stratified analyses, which guide more targeted prevention strategies. Meanwhile, the study also has several limitations: Firstly, as an ecological study, the association between cold spells and COPD hospitalizations does not imply causality. Secondly, different socio-economic status or other factors on an individual level might be confounding factors and were not considered in the association. Thirdly, the meteorological variables and AQI were all from monitoring stations, not reflecting the individual level of exposures. Moreover, people are more likely to stay indoors with heating system during extremely cold days in northern China, so inevitable exposure measurement errors may lead to bias. Lastly, the data from only one city weakens the extrapolation validity of the study.

For peer review only

CONCLUSION

Our study demonstrates that short-term exposure to cold spells is associated with an increased risk of COPD hospitalizations. The cumulative effects increased with the intensities and the durations of cold spells. The elderly are more vulnerable to COPD hospitalizations during cold spell periods. These findings provide scientific foundations for comprehensive public health strategies to reduce cold spell-related COPD hospitalizations in Beijing, China.

Contributors: YL and YC are joint first author and designed the study. ZF obtained the

For peer review only - http://bmjopen.bmj.com/site/about/guidelines.xhtml

original data and funding. DK, XL, JF, YZ and ZC preprocessed the data. YL, YC, and YZ analyzed the data. YL and YC drafted the manuscript. XZ, KX, CJ and ZF reviewed and edited the manuscript. All authors have read and approved the final manuscript. ZF is the study guarantor.

Funding: This work was supported by National Key Research and Development Plan [2017YFC0211703], National 973 Project [2015CB553400], Chinese Academy of Medical Sciences (CAMS) Initiative for Innovative Medicine [CAMS 2016ZX310181-5/4, CAMS 2017-I2M-2-001], and National Natural Science Foundation [91643208, 41450006].

Competing interests: None declared.

Ethical approval: This study was approved by the ethical review committee of Peking Union Medical College Hospital.

Data sharing statement: No additional data are available.

For peer review only

REFERENCES

1 IPCC, 2014. *Climate Change 2014: Synthesis Report. Contribution of Working Groups I, II and III to the Fifth Assessment Report of the Intergovernmental Panel on Climate Change* [Core Writing Team, R.K. Pachauri and L.A. Meyer (eds.)]. IPCC, Geneva, Switzerland, 151 pp.

2 Cohen J, Screen JA, Furtado JC*, et al.* Recent Arctic amplification and extreme mid-latitude weather. *Nature Geoscience* 2014;**7**:627-37.

3 Zhang J, Tian W, Chipperfield MP*, et al.* Persistent shift of the Arctic polar vortex towards the Eurasian continent in recent decades. *Nature Climate Change* 2016;**6**:1094-9.

4 Chen J, Yang J, Zhou M*, et al.* Cold spell and mortality in 31 Chinese capital cities: Definitions, vulnerability and implications. *Environment international* 2019;**128**:271-8.

5 Lee W, Choi HM, Lee JY*, et al.* Temporal changes in mortality impacts of heat wave and cold spell in Korea and Japan. *Environment international* 2018;**116**:136-46.

6 Han J, Liu S, Zhang J*, et al.* The impact of temperature extremes on mortality: a time-series study in Jinan, China. *BMJ open* 2017;**7**:e014741.

7 Rabe KF, Watz H. Chronic obstructive pulmonary disease. *The Lancet* 2017;**389**:1931-40.

8 Lopez-Campos JL, Tan W, Soriano JB. Global burden of COPD. *Respirology* 2016;**21**:14-23.

9 Donaldson GC, Goldring JJ, Wedzicha JA. Influence of season on exacerbation characteristics in patients with COPD. *Chest* 2012;**141**:94-100.

10 McCormack MC, Paulin LM, Gummerson CE*, et al.* Colder temperature is associated with increased COPD morbidity. *Eur Respir J* 2017;**49**.

11 Chaturvedi S, Tseng C-M, Chen Y-T*, et al.* The Effect of Cold Temperature on Increased Exacerbation of Chronic Obstructive Pulmonary Disease: A Nationwide Study. *PloS one* 2013;**8**.

12 Monteiro A, Carvalho V, Gois J*, et al.* Use of "Cold Spell" indices to quantify excess chronic obstructive pulmonary disease (COPD) morbidity during winter (November to March 2000-2007): case study in Porto. *International journal of biometeorology* 2013;**57**:857-70.

13 Wang C, Xu J, Yang L*, et al.* Prevalence and risk factors of chronic obstructive pulmonary disease in China (the China Pulmonary Health [CPH] study): a national cross-sectional study. *The Lancet* 2018;**391**:1706-17.

14 Xie H, Yao Z, Zhang Y*, et al.* Short-term effects of the 2008 cold spell on mortality in three subtropical cities in Guangdong Province, China. *Environmental health perspectives* 2013;**121**:210-6.

15 Zhou B, Gu L, Ding Y*, et al.* The Great 2008 Chinese Ice Storm: Its Socioeconomic–Ecological Impact and Sustainability Lessons Learned. *Bulletin of the American Meteorological Society* 2011;**92**:47-60.

16 Ban J, Huang L, Chen C*, et al.* Integrating new indicators of predictors that shape the

For peer review only

For peer review only - http://bmjopen.bmj.com/site/about/guidelines.xhtml

public's perception of local extreme temperature in China. *The Science of the total environment* 2017;**579**:529-36.

17 Wang L, Liu T, Hu M*, et al.* The impact of cold spells on mortality and effect modification by cold spell characteristics. *Scientific reports* 2016;**6**:38380.

18 Gao J, Yu F, Xu Z*, et al.* The association between cold spells and admissions of ischemic stroke in Hefei, China: Modified by gender and age. *The Science of the total environment* 2019;**669**:140-7.

19 Wedzicha JA, Seemungal TAR. COPD exacerbations: defining their cause and prevention. *The Lancet* 2007;**370**:786-96.

20 Cowling BJ, Wong IO, Ho LM*, et al.* Methods for monitoring influenza surveillance data. *Int J Epidemiol* 2006;**35**:1314-21.

21 Song X, Wang S, Li T*, et al.* The impact of heat waves and cold spells on respiratory emergency department visits in Beijing, China. *The Science of the total environment* 2018;**615**:1499-505.

22 Guo Y, Jiang F, Peng L*, et al.* The association between cold spells and pediatric outpatient visits for asthma in Shanghai, China. *PloS one* 2012;**7**:e42232.

23 Gasparrini A. Modeling exposure-lag-response associations with distributed lag non-linear models. *Stat Med* 2014;**33**:881-99.

24 Cheng Q, Wang X, Wei Q*, et al.* The short-term effects of cold spells on pediatric outpatient admission for allergic rhinitis in Hefei, China. *The Science of the total environment* 2019;**664**:374-80.

25 Liang Z, Wang P, Zhao Q*, et al.* Effect of the 2008 cold spell on preterm births in two subtropical cities of Guangdong Province, Southern China. *The Science of the total environment* 2018;**642**:307-13.

26 de'Donato FK, Leone M, Noce D*, et al.* The impact of the February 2012 cold spell on health in Italy using surveillance data. *PloS one* 2013;**8**:e61720.

27 Lin Z, Gu Y, Liu C*, et al.* Effects of ambient temperature on lung function in patients with chronic obstructive pulmonary disease: A time-series panel study. *The Science of the total environment* 2018;**619-620**:360-5.

28 Donaldson GC, Seemungal T, Jeffries DJ*, et al.* Effect of temperature on lung function and symptoms in chronic obstructive pulmonary disease. *Eur Respir J* 1999;**13**:844-9.

29 Koskela HO, Koskela AK, Tukiaineu HO. Bronchoconstriction due to cold weather in COPD. The roles of direct airway effects and cutaneous reflex mechanisms. *Chest* 1996;**110**:632-6.

30 Shephard RJ, Shek PN. Cold exposure and immune function. *Can J Physiol Pharmacol* 1998;**76**:828-36.

31 Lowen AC, Mubareka S, Steel J*, et al.* Influenza virus transmission is dependent on relative humidity and temperature. *PLoS pathogens* 2007;**3**:1470-6.

32 Li M, Li Q, Yang G*, et al.* Cold temperature induces mucin hypersecretion from normal

For peer review only - http://bmjopen.bmj.com/site/about/guidelines.xhtml

human bronchial epithelial cells in vitro through a transient receptor potential melastatin 8 (TRPM8)-mediated mechanism. *J Allergy Clin Immunol* 2011;**128**:626-34.e1-5.

33 Juan Y, Haiqiao W, Xie W*, et al.* Cold-inducible RNA-binding protein mediates airway inflammation and mucus hypersecretion through a post-transcriptional regulatory mechanism under cold stress. *Int J Biochem Cell Biol* 2016;**78**:335-48.

34 Chung Y, Noh H, Honda Y*, et al.* Temporal Changes in Mortality Related to Extreme Temperatures for 15 Cities in Northeast Asia: Adaptation to Heat and Maladaptation to Cold. *American journal of epidemiology* 2017;**185**:907-13.

35 Ma W, Yang C, Chu C*, et al.* The impact of the 2008 cold spell on mortality in Shanghai, China. *International journal of biometeorology* 2013;**57**:179-84.

36 Tseng CM, Chen YT, Ou SM*, et al.* The effect of cold temperature on increased exacerbation of chronic obstructive pulmonary disease: a nationwide study. *PloS one* 2013;**8**:e57066.

37 Goodwin JS. Decreased immunity and increased morbidity in the elderly. *Nutr Rev* 1995;**53**:S41-4; discussion S4-6.

38 Donaldson GC, Ermakov SP, Komarov YM*, et al.* Cold related mortalities and protection against cold in Yakutsk, eastern Siberia: observation and interview study. *BMJ* 1998;**317**:978-82.

39 Qiu H, Tan K, Long F*, et al.* The Burden of COPD Morbidity Attributable to the Interaction between Ambient Air Pollution and Temperature in Chengdu, China. *International journal of environmental research and public health* 2018;**15**.

40 Huynen MM, Martens P, Schram D*, et al.* The impact of heat waves and cold spells on mortality rates in the Dutch population. *Environmental health perspectives* 2001;**109**:463-70.

For peer review only

For peer review only - http://bmjopen.bmj.com/site/about/guidelines.xhtml

Table 1 Statistic summary of daily hospitalizations for COPD, meteorological variables and air quality index in cold seasons (November-March) in Beijing, China, 2012–2016.

	Total	Mean (SD)	Min	P25	Median	P75	Max
COPD cases	84571	111.7(41.0)	22.0	78.0	113.0	141.0	226.0
Gender							
Male	53829	71.1(25.8)	12.0	49.0	75.0	89.0	158.0
Female	30742	40.6(17.7)	7.0	27.0	39.0	53.0	110.0
Age							
0-64 years old	13600	18.0(8.3)	2.0	12.0	18.0	23.0	43.0
≥65 years old	70971	93.8(34.3)	19.0	67.0	95.0	118.0	189.0
Mean temperature (℃)	/	0.9(5.4)	-16.0	-3.0	0.0	4.0	18.0
Relative humidity (%)	/	46.6(20.4)	8.0	30.0	43.0	61.0	98.0
Air pressure (hPa)	/	1025.3(6.5)	1005.0	1021.0	1026.0	1030.0	1044.0
Air quality index	/	126.3(89.2)	17.0	61.0	97.0	170.0	485.0

COPD, chronic obstructive pulmonary disease; P25, the 25th percentile; P75, the 75th percentile; SD, standard deviation.

For peer review only - http://bmjopen.bmj.com/site/about/guidelines.xhtml

Table 2 Overview information of cold spells under different definitions

Model	Temperature threshold	Duration	Cold spell episodes	Cold spell days	Non-cold spell days	Q-AIC value
1	≤10% (-6℃)	≥2d	17	77	680	7819.6
2	≤10% (-6℃)	≥3d	11	65	692	7799.7
3	≤10% (-6℃)	≥4d	6	50	707	7769.8
4	≤5% (-7℃)	≥2d	8	37	720	7794.3
5	≤5% (-7℃)	≥3d	5	31	726	7786.6
6	≤5% (-7℃)	≥4d	3	25	732	7782.5
7	≤3% (-8℃)	≥2d	7	23	734	7786.5
8	≤3% (-8℃)	≥3d	5	19	738	7789.8
9	≤3% (-8℃)	≥4d	2	10	747	7804.4

Q-AIC, quasi-Poisson Akaike Information Criterion.

For peer review only

For peer review only - http://bmjopen.bmj.com/site/about/guidelines.xhtml

Table 3 Cumulative relative risk of cold spells under different definitions on COPD hospitalizations of the total population in Beijing, 2012-2016.

Definition	CRR (95% CI)			
	Lag0	Lag0-7	Lag0-14	Lag0-21
≤10% & ≥2d	1.030 (1.002-1.058)*	1.200 (1.087-1.326)*	1.278 (1.134-1.439)*	1.236 (1.055-1.438)*
≤10% & ≥3d	1.027 (1.000-1.054)*	1.188 (1.084-1.303)*	1.299 (1.166-1.448)*	1.353 (1.161-1.577)*
≤10% &≥4d	1.042 (1.013-1.072)*	1.249 (1.136-1.374)*	1.343 (1.206-1.496)*	1.394 (1.193-1.630)*
≤5% & ≥2d	1.012 (0.980-1.045)	1.173 (1.055-1.304)*	1.344 (1.134-1.439)*	1.354 (1.141-1.608)*
≤5% & ≥3d	1.031 (1.000-1.063)*	1.235 (1.114-1.369)*	1.381 (1.220-1.563)*	1.428 (1.206-1.692)*
≤5% & ≥4d	1.042 (1.006-1.079)*	1.268 (1.132-1.421)*	1.404 (1.234-1.598)*	1.511 (1.262-1.809)*
≤3% & ≥2d	0.982 (0.944-1.021)	1.113 (0.960-1.290)	1.412 (1.171-1.703)*	1.444 (1.113-1.873)*
≤3% & ≥3d	1.009 (0.970-1.049)	1.217 (1.051-1.411)*	1.525 (1.264-1.841)*	1.659 (1.278-2.154)*
≤3% & ≥4d	0.992 (0.951-1.035)	1.201 (0.999-1.443)	1.644 (1.261-2.145)*	1.889 (1.315-2.712)*

CI, confidence interval; CRR, cumulative relative risk.

*p<0.05.

For peer review only

For peer review only - http://bmjopen.bmj.com/site/about/guidelines.xhtml

Table 4 Cumulative relative risk of cold spells on COPD hospitalizations stratified by gender and age in Beijing, 2012-2016.

Subgroup	CRR (95% CI)			
	Lag0	Lag0-7	Lag0-14	Lag0-21
Male	1.042 (1.011-1.074)*	1.243 (1.123-1.375)*	1.316 (1.173-1.477)*	1.342 (1.136-1.586)*
Female	1.041 (1.005-1.077)*	1.257 (1.119-1.411)*	1.383 (1.215-1.574)*	1.476 (1.211-1.783)*
Age<65	1.017 (0.972-1.064)	1.120 (0.963-1.303)	1.159 (0.977-1.376)	1.107 (0.862-1.422)
Age≥65	1.046 (1.017-1.077)*	1.275 (1.158-1.404)*	1.382 (1.240-1.540)*	1.456 (1.244-1.705)*

CI, confidence interval; CRR, cumulative relative risk.

*p<0.05.

For peer review only

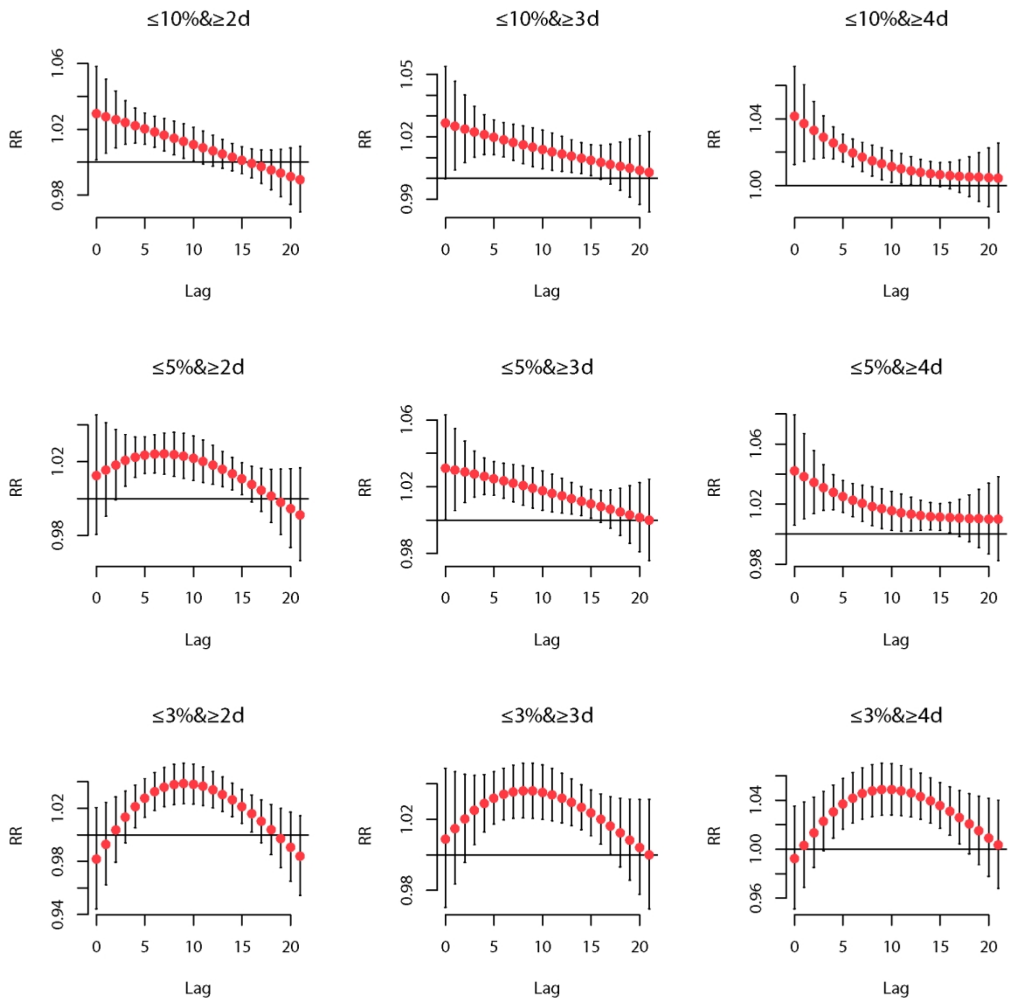

Figure 1 Lag-response relationships between cold spells under different definitions and AECOPD hospitalizations of the total population in Beijing, 2012-2016.

161x159mm (300 x 300 DPI)

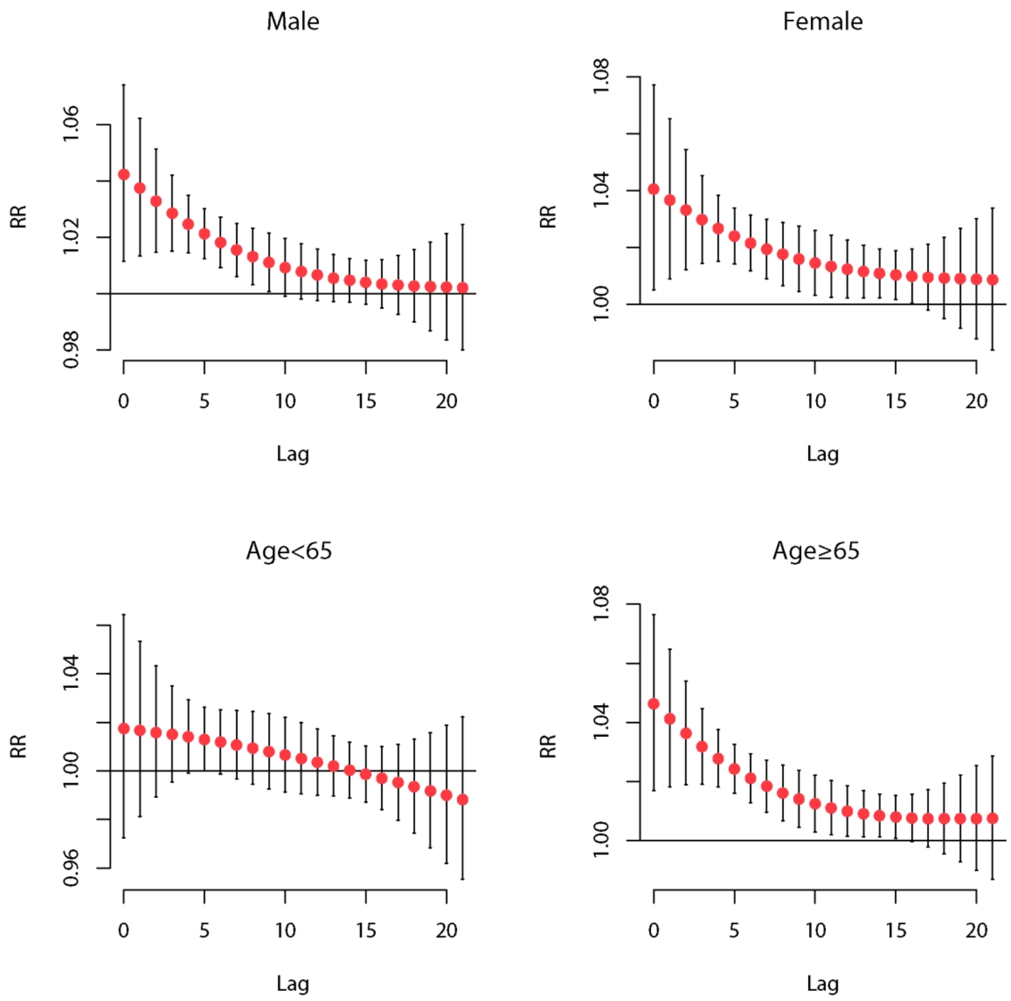

Figure 2 Lag-response relationships between cold spells and AECOPD hospitalizations stratified by gender and age in Beijing, 2012-2016.

159x157mm (300 x 300 DPI)

Supplementary Materials

Supplementary Tables

Table S1 The cumulative effects of cold spells under the optimal definition using different degrees of freedom for lag dimension in the DLM model

Table S2 The cumulative effects of cold spells under the optimal definition using different degrees of freedom for time per year in the DLM model

Table S3 The cumulative effects of cold spells under the optimal definition using different degrees of freedom for relative humidity in the DLM model

Table S4 The cumulative effects of cold spells under the optimal definition using different degrees of freedom for air pressure in the DLM model

Table S5 The cumulative effects of cold spells under the optimal definition using different degrees of freedom for air quality index in the DLM model

For peer review only

Table S1 The cumulative effects of cold spells under the optimal definition using different degrees of freedom for lag dimension in the DLM model

df for lag dimension	Group	CRR (95% CI)			
		Lag0	Lag0-7	Lag0-14	Lag0-21
3[a]	Total	1.042 (1.013-1.072)*	1.249 (1.136-1.374)*	1.343 (1.206-1.496)*	1.394 (1.193-1.630)*
	Male	1.042 (1.011-1.074)*	1.243 (1.123-1.375)*	1.316 (1.173-1.477)*	1.342 (1.136-1.586)*
	Female	1.041 (1.005-1.077)*	1.257 (1.119-1.411)*	1.383 (1.215-1.574)*	1.476 (1.211-1.783)*
	Age<65	1.017 (0.972-1.064)	1.120 (0.963-1.303)	1.159 (0.977-1.376)	1.107 (0.862-1.422)
	Age≥65	1.046 (1.017-1.077)*	1.275 (1.158-1.404)*	1.382 (1.240-1.540)*	1.456 (1.244-1.705)*
4	Total	0.989 (0.926-1.057)	1.265 (1.149-1.392)*	1.311 (1.173-1.465)*	1.382 (1.182-1.615)*
	Male	0.992 (0.925-1.064)	1.257 (1.135-1.393)*	1.285 (1.141-1.448)*	1.331 (1.126-1.573)*
	Female	0.983 (0.906-1.067)	1.274 (1.133-1.432)*	1.347 (1.178-1.540)*	1.462 (1.210-1.766)*
	Age<65	0.919 (0.827-1.022)	1.151 (0.988-1.340)	1.106 (0.926-1.320)	1.091 (0.850-1.401)
	Age≥65	1.003 (0.938-1.073)	1.288 (1.169-1.419)*	1.355 (1.211-1.515)*	1.446 (1.235-1.692)*
5	Total	1.001 (0.888-1.128)	1.271 (1.150-1.404)*	1.313 (1.174-1.468)*	1.389 (1.185-1.628)*
	Male	0.999 (0.880-1.136)	1.261 (1.134-1.403)*	1.287 (1.141-1.451)*	1.336 (1.127-1.583)*
	Female	1.004 (0.868-1.161)	1.285 (1.137-1.451)*	1.350 (1.180-1.545)*	1.474 (1.216-1.787)*
	Age<65	0.892 (0.737-1.080)	1.143 (0.975-1.341)	1.102 (0.922-1.317)	1.084 (0.840-1.398)
	Age≥65	1.023 (0.907-1.155)	1.297 (1.172-1.434)*	1.358 (1.213-1.520)*	1.456 (1.240-1.710)*

CI, confidence interval; df, degree of freedom; RR, relative risk.

*P<0.05.

[a]Used in the study.

For peer review only

Table S2 The cumulative effects of cold spells under the optimal definition using different degrees of freedom for time per year in the DLM model

df for time per year	Group	CRR (95% CI)			
		Lag0	Lag0-7	Lag0-14	Lag0-21
3[a]	Total	1.042 (1.013-1.072)*	1.249 (1.136-1.374)*	1.343 (1.206-1.496)*	1.394 (1.193-1.630)*
	Male	1.042 (1.011-1.074)*	1.243 (1.123-1.375)*	1.316 (1.173-1.477)*	1.342 (1.136-1.586)*
	Female	1.041 (1.005-1.077)*	1.257 (1.119-1.411)*	1.383 (1.215-1.574)*	1.476 (1.211-1.783)*
	Age<65	1.017 (0.972-1.064)	1.120 (0.963-1.303)	1.159 (0.977-1.376)	1.107 (0.862-1.422)
	Age⩾65	1.046 (1.017-1.077)*	1.275 (1.158-1.404)*	1.382 (1.240-1.540)*	1.456 (1.244-1.705)*
4	Total	1.023 (0.992-1.055)	1.150 (1.033-1.280)*	1.218 (1.078-1.376)*	1.233 (1.036-1.467)*
	Male	1.029 (0.997-1.063)	1.173 (1.045-1.316)*	1.226 (1.074-1.399)*	1.225 (1.015-1.480)*
	Female	1.011 (0.974-1.050)	1.107 (0.971-1.262)	1.196 (1.034-1.385)*	1.232 (0.998-1.520)
	Age<65	1.010 (0.961-1.061)	1.077 (0.906-1.280)	1.105 (0.907-1.345)	1.056 (0.795-1.401)
	Age⩾65	1.026 (0.995-1.058)	1.166 (1.046-1.300)*	1.244 (1.100-1.406)*	1.272 (1.067-1.517)*
5	Total	1.022 (0.990-1.056)	1.144 (1.008-1.298)*	1.208 (1.031-1.415)*	1.222 (0.978-1.526)
	Male	1.030 (0.994-1.066)	1.171 (1.021-1.343)*	1.222 (1.027-1.452)*	1.223 (0.960-1.559)
	Female	1.009 (0.970-1.050)	1.093 (0.938-1.275)	1.174 (0.972-1.418)	1.203 (0.923-1.569)
	Age<65	1.011 (0.959-1.066)	1.082 (0.883-1.327)	1.127 (0.874-1.453)	1.127 (0.788-1.612)
	Age⩾65	1.025 (0.992-1.059)	1.158 (1.019-1.316)*	1.227 (1.045-1.440)*	1.245 (0.994-1.559)

CI, confidence interval; df, degree of freedom; RR, relative risk.

*P<0.05.

[a]Used in the study.

For peer review only

Table S3 The cumulative effects of cold spells under the optimal definition using different degrees of freedom for relative humidity in the DLM model

df for relative humidity	Group	CRR (95% CI)			
		Lag0	Lag0-7	Lag0-14	Lag0-21
3[a]	Total	1.042 (1.013-1.072)[*]	1.249 (1.136-1.374)[*]	1.343 (1.206-1.496)[*]	1.394 (1.193-1.630)[*]
	Male	1.042 (1.011-1.074)[*]	1.243 (1.123-1.375)[*]	1.316 (1.173-1.477)[*]	1.342 (1.136-1.586)[*]
	Female	1.041 (1.005-1.077)[*]	1.257 (1.119-1.411)[*]	1.383 (1.215-1.574)[*]	1.476 (1.211-1.783)[*]
	Age<65	1.017 (0.972-1.064)	1.120 (0.963-1.303)	1.159 (0.977-1.376)	1.107 (0.862-1.422)
	Age≥65	1.046 (1.017-1.077)[*]	1.275 (1.158-1.404)[*]	1.382 (1.240-1.540)[*]	1.456 (1.244-1.705)[*]
4	Total	1.041 (1.012-1.071)[*]	1.248 (1.135-1.373)[*]	1.343 (1.205-1.495)[*]	1.392 (1.190-1.629)[*]
	Male	1.042 (1.011-1.073)[*]	1.240 (1.120-1.372)[*]	1.314 (1.170-1.475)[*]	1.336 (1.130-1.580)[*]
	Female	1.041 (1.006-1.078)[*]	1.259 (1.120-1.414)[*]	1.385 (1.217-1.578)[*]	1.482 (1.225-1.792)[*]
	Age<65	1.017 (0.972-1.064)	1.119 (0.961-1.302)	1.158 (0.975-1.375)	1.105 (0.859-1.420)
	Age≥65	1.046 (1.017-1.076)[*]	1.274 (1.157-1.403)[*]	1.381 (1.239-1.540)[*]	1.455 (1.242-1.704)[*]
5	Total	1.041 (1.012-1.071)[*]	1.244 (1.131-1.369)[*]	1.337 (1.200-1.490)[*]	1.383 (1.182-1.619)[*]
	Male	1.041 (1.010-1.073)[*]	1.236 (1.116-1.368)[*]	1.307 (1.164-1.468)[*]	1.327 (1.121-1.569)[*]
	Female	1.040 (1.005-1.077)[*]	1.256 (1.117-1.411)[*]	1.381 (1.212-1.574)[*]	1.474 (1.218-1.785)[*]
	Age<65	1.015 (0.970-1.062)	1.111 (0.955-1.293)	1.148 (0.967-1.363)	1.089 (0.847-1.400)
	Age≥65	1.046 (1.016-1.076)[*]	1.271 (1.154-1.400)[*]	1.377 (1.234-1.536)[*]	1.448 (1.235-1.697)[*]

CI, confidence interval; df, degree of freedom; RR, relative risk.

[*]$P<0.05$.

[a]Used in the study.

For peer review only

Table S4 The cumulative effects of cold spells under the optimal definition using different degrees of freedom for air pressure in the DLM model

df for air pressure	Group	CRR (95% CI)			
		Lag0	Lag0-7	Lag0-14	Lag0-21
3[a]	Total	1.042 (1.013-1.072)*	1.249 (1.136-1.374)*	1.343 (1.206-1.496)*	1.394 (1.193-1.630)*
	Male	1.042 (1.011-1.074)*	1.243 (1.123-1.375)*	1.316 (1.173-1.477)*	1.342 (1.136-1.586)*
	Female	1.041 (1.005-1.077)*	1.257 (1.119-1.411)*	1.383 (1.215-1.574)*	1.476 (1.211-1.783)*
	Age<65	1.017 (0.972-1.064)	1.120 (0.963-1.303)	1.159 (0.977-1.376)	1.107 (0.862-1.422)
	Age≥65	1.046 (1.017-1.077)*	1.275 (1.158-1.404)*	1.382 (1.240-1.540)*	1.456 (1.244-1.705)*
4	Total	1.042 (1.012-1.071)*	1.248 (1.134-1.373)*	1.342 (1.205-1.495)*	1.393 (1.192-1.629)*
	Male	1.042 (1.011-1.074)*	1.240 (1.120-1.373)*	1.313 (1.170-1.475)*	1.340 (1.134-1.584)*
	Female	1.041 (1.005-1.078)*	1.259 (1.120-1.414)*	1.385 (1.216-1.578)*	1.478 (1.223-1.786)*
	Age<65	1.018 (0.973-1.065)	1.123 (0.965-1.306)	1.162 (0.978-1.380)	1.109 (0.863-1.425)
	Age≥65	1.046 (1.016-1.076)*	1.273 (1.156-1.402)*	1.380 (1.238-1.539)*	1.455 (1.242-1.704)*
5	Total	1.041 (1.012-1.071)*	1.247 (1.133-1.373)*	1.342 (1.204-1.495)*	1.393 (1.191-1.629)*
	Male	1.042 (1.011-1.074)*	1.240 (1.120-1.374)*	1.314 (1.170-1.476)*	1.341 (1.134-1.585)*
	Female	1.041 (1.005-1.077)*	1.257 (1.118-1.413)*	1.383 (1.214-1.576)*	1.475 (1.220-1.784)*
	Age<65	1.019 (0.974-1.067)	1.129 (0.970-1.314)	1.168 (0.984-1.388)	1.115 (0.868-1.433)
	Age≥65	1.046 (1.016-1.076)*	1.271 (1.154-1.401)*	1.378 (1.235-1.537)*	1.452 (1.240-1.701)*

CI, confidence interval; df, degree of freedom; RR, relative risk.
*$P<0.05$.
[a]Used in the study.

For peer review only

Table S5 The cumulative effects of cold spells under the optimal definition using different degrees of freedom for air quality index in the DLM model

df for AQI	Group	CRR (95% CI)			
		Lag0	Lag0-7	Lag0-14	Lag0-21
3[a]	Total	1.042 (1.013-1.072)*	1.249 (1.136-1.374)*	1.343 (1.206-1.496)*	1.394 (1.193-1.630)*
	Male	1.042 (1.011-1.074)*	1.243 (1.123-1.375)*	1.316 (1.173-1.477)*	1.342 (1.136-1.586)*
	Female	1.041 (1.005-1.077)*	1.257 (1.119-1.411)*	1.383 (1.215-1.574)*	1.476 (1.211-1.783)*
	Age<65	1.017 (0.972-1.064)	1.120 (0.963-1.303)	1.159 (0.977-1.376)	1.107 (0.862-1.422)
	Age≥65	1.046 (1.017-1.077)*	1.275 (1.158-1.404)*	1.382 (1.240-1.540)*	1.456 (1.244-1.705)*
4	Total	1.042 (1.013-1.072)*	1.249 (1.135-1.374)*	1.342 (1.205-1.495)*	1.393 (1.192-1.629)*
	Male	1.042 (1.012-1.074)*	1.242 (1.123-1.375)*	1.315 (1.171-1.476)*	1.342 (1.135-1.586)*
	Female	1.041 (1.005-1.077)*	1.257 (1.119-1.412)*	1.382 (1.214-1.573)*	1.475 (1.221-1.783)*
	Age<65	1.018 (0.973-1.065)	1.120 (0.963-1.303)	1.157 (0.974-1.373)	1.106 (0.861-1.421)
	Age≥65	1.046 (1.017-1.077)*	1.275 (1.158-1.404)*	1.381 (1.239-1.539)*	1.456 (1.243-1.704)*
5	Total	1.042 (1.013-1.071)*	1.249 (1.136-1.373)*	1.347 (1.210-1.500)*	1.405 (1.202-1.643)*
	Male	1.042 (1.011-1.074)*	1.242 (1.123-1.373)*	1.320 (1.177-1.480)*	1.355 (1.148-1.601)*
	Female	1.041 (1.005-1.077)*	1.257 (1.119-1.412)*	1.385 (1.216-1.577)*	1.482 (1.226-1.792)*
	Age<65	1.018 (0.973-1.065)	1.120 (0.963-1.303)	1.161 (0.978-1.378)	1.116 (0.869-1.433)
	Age≥65	1.046 (1.017-1.076)*	1.275 (1.159-1.403)*	1.386 (1.243-1.545)*	1.468 (1.254-1.718)*

AQI, air quality index; CI, confidence interval; df, degree of freedom; RR, relative risk.

*P<0.05.

[a]Used in the study.

For peer review only

BMJ Open

The short-term effects of cold spells on hospitalizations for acute exacerbation of chronic obstructive pulmonary disease: a time-series study in Beijing, China

Journal:	*BMJ Open*
Manuscript ID	bmjopen-2020-039745.R1
Article Type:	Original research
Date Submitted by the Author:	10-Sep-2020
Complete List of Authors:	Liu, Yanbo; Peking Union Medical College Hospital, Department of Medicine<br>Chen, Yuxiong; Peking Union Medical College Hospital, Department of Medicine<br>Kong, Dehui; Peking Union Medical College Hospital, Department of Medicine<br>Liu, Xiaole; Peking Union Medical College Hospital, Department of Medicine<br>Fu, Jia; Peking Union Medical College Hospital, Department of Medicine<br>Zhang, Yongqiao; Peking Union Medical College Hospital, Department of Medicine<br>Zhao, Yakun; Peking Union Medical College Hospital, Department of Medicine<br>Chang, Zhen'ge; Peking Union Medical College Hospital, Department of Medicine<br>Zhao, Xiaoyi; Peking Union Medical College Hospital, Department of Physiotherapy<br>Xu, Kaifeng; Peking Union Medical College Hospital, Department of Medicine<br>Jiang, Chengyu; Chinese Academy of Medical Sciences and Peking Union Medical College State Key Laboratory of Medical Molecular Biology<br>Fan, Zhongjie; Peking Union Medical College Hospital, Department of Medicine
**Primary Subject Heading**:	Public health
Secondary Subject Heading:	Respiratory medicine, Epidemiology
Keywords:	Epidemiology < TROPICAL MEDICINE, PUBLIC HEALTH, Chronic airways disease < THORACIC MEDICINE

SCHOLARONE™
Manuscripts

I, the Submitting Author has the right to grant and does grant on behalf of all authors of the Work (as defined in the below author licence), an exclusive licence and/or a non-exclusive licence for contributions from authors who are: i) UK Crown employees; ii) where BMJ has agreed a CC-BY licence shall apply, and/or iii) in accordance with the terms applicable for US Federal Government officers or employees acting as part of their official duties; on a worldwide, perpetual, irrevocable, royalty-free basis to BMJ Publishing Group Ltd ("BMJ") its licensees and where the relevant Journal is co-owned by BMJ to the co-owners of the Journal, to publish the Work in this journal and any other BMJ products and to exploit all rights, as set out in our licence.

The Submitting Author accepts and understands that any supply made under these terms is made by BMJ to the Submitting Author unless you are acting as an employee on behalf of your employer or a postgraduate student of an affiliated institution which is paying any applicable article publishing charge ("APC") for Open Access articles. Where the Submitting Author wishes to make the Work available on an Open Access basis (and intends to pay the relevant APC), the terms of reuse of such Open Access shall be governed by a Creative Commons licence – details of these licences and which Creative Commons licence will apply to this Work are set out in our licence referred to above.

Other than as permitted in any relevant BMJ Author's Self Archiving Policies, I confirm this Work has not been accepted for publication elsewhere, is not being considered for publication elsewhere and does not duplicate material already published. I confirm all authors consent to publication of this Work and authorise the granting of this licence.

For peer review only

The short-term effects of cold spells on hospitalizations for acute exacerbation of chronic obstructive pulmonary disease: a time-series study in Beijing, China

Authors: Yanbo Liu[1#], Yuxiong Chen[1#], Dehui Kong[1], Xiaole Liu[1], Jia Fu[1], Yongqiao Zhang[1], Yakun Zhao[1], Zhen'ge Chang[1], Xiaoyi Zhao[2], Kaifeng Xu[1], Chengyu Jiang[3], Zhongjie Fan[1*]

Affiliation and Address:

[1]Department of Medicine, Peking Union Medical College Hospital, Peking Union Medical College & Chinese Academy of Medical Sciences, No.1 Shuaifuyuan Wangfujing Dongcheng District, Beijing 100730, China.

[2]Department of Physiotherapy, Peking Union Medical College Hospital, Peking Union Medical College & Chinese Academy of Medical Sciences, No.1 Shuaifuyuan Wangfujing Dongcheng District, Beijing 100730, China.

[3]The State Key Laboratory of Medical Molecular Biology, Institute of Basic Medical Sciences, Chinese Academy of Medical Sciences, Department of Biochemistry, Peking Union Medical College, Beijing 100005, China.

[#]Yanbo Liu and Yuxiong Chen contributed equally to this work.

[*]**Corresponding author:**

Zhongjie Fan, Department of Medicine, Peking Union Medical College Hospital, Peking Union Medical College & Chinese Academy of Medical Sciences, No.1 Shuaifuyuan Wangfujing Dongcheng District, Beijing 100730, China (Fanzhongjie@pumch.cn)

Keywords: cold spells, acute exacerbation of chronic obstructive pulmonary disease, hospitalizations, intensity and duration

For peer review only

For peer review only - http://bmjopen.bmj.com/site/about/guidelines.xhtml

ABSTRACT

Objectives: Our work aimed at exploring the relationship between cold spells and acute exacerbation of chronic obstructive pulmonary disease (AECOPD) hospitalizations in Beijing, China, and assessing the moderating effects of the intensities and the durations of cold spells, as well as identifying the vulnerable.

Design: A time-series study.

Setting: We obtained time-series data of AECOPD hospitalizations, meteorological variables and air quality index in Beijing, China during 2012–2016.

Participants: All AECOPD hospitalizations among permanent residents in Beijing, China in cold seasons (November to March) during 2012-2016 were included. (n=84,571).

Primary and secondary outcome measures: A quasi-Poisson regression with a distributed lag model was fitted to investigate the short-term effects of cold spells on AECOPD hospitalizations by comparing the counts of AECOPD admissions during cold spell days with those during non-cold spell days.

Results: Cold spells under different definitions were associated with increased risks of AECOPD hospitalizations, with the maximum cumulative relative risk (CRR) over three weeks (lag0-21). The cumulative effects at lag0-21 increased with the intensities and the durations of cold spells. Under the optimal definition, the most significant single-day relative risk (RR) was found on the days of cold spells (lag0) with RR=1.042 (95%CI: 1.013, 1.072), and the CRR at lag0-21 was 1.394 (95%CI: 1.193, 1.630). The elderly (aged≥65) were more vulnerable to the effects of cold spells on AECOPD hospitalizations.

Conclusion: Cold spells are associated with increased AECOPD hospitalizations in

For peer review only - http://bmjopen.bmj.com/site/about/guidelines.xhtml

Beijing, with the cumulative effects increased with their intensities and durations. The elderly are at particular risk of AECOPD hospitalizations triggered by cold spells.

Strengths and limitations of this study:

- This study was the first to examine the association between cold spells and AECOPD hospitalizations in China.
- The study assessed the effects of cold spells under different definitions on AECOPD hospitalizations to find out the optimal cold spell definition on the issue.
- The ecological design cannot imply causality definitely, while limited information on individual-level factors and inevitable exposure measurement errors may lead to bias.
- The data from one specific city limited the extrapolation of the findings.

INTRODUCTION

The Intergovernmental Panel on Climate Change (IPCC) has predicted that human activities and global climate change cause variations in frequency, intensity and duration of many extreme weather events, including heatwaves and cold spells.[1] Although the amount of cold spells may decrease over most land areas due to global warming, a few recent studies found that the persistent shift of the Arctic polar vortex and Arctic amplification associated with global warming could lead to increased extremely cold events in mid-latitudes.[2, 3]. Over the last few years, the impacts of cold spells on human health have gained growing attention from the public. Many studies have reported positive relationships between cold spells and mortality[4-6] while the impacts of cold spells on hospital visits or admissions are under-examined.

Chronic obstructive pulmonary disease (COPD) is one of the common respiratory

For peer review only - http://bmjopen.bmj.com/site/about/guidelines.xhtml

diseases characterized by poorly reversible limitation of airflow.[7] Owing to its high prevalence, morbidity, mortality and economic burden globally, COPD has been an important public health concern and will remain a huge challenge for healthcare practitioners in the foreseeable future.[8] Thus, it is crucial to identify the risk factors of COPD to improve strategies on prevention and intervention. Given the projected climate change, extreme temperature events potentially pose threats to COPD patients. Many epidemiological studies have indicated that COPD has higher rates of exacerbation and hospitalization with lower temperatures.[9-13] We hypothesized that cold spells, defined as prolonged periods of extremely cold weather, may be more detrimental to COPD patients, and could cause more hospitalizations for acute exacerbations (AECOPD).[14] However, few studies have been carried out on the association between cold spells and AECOPD hospitalizations.[15]

As the world’s largest country by population, China shoulders the enormous burden of COPD. A national cross-sectional study from 2012 to 2015 showed that the prevalence of COPD among Chinese adults aged 20 years and older was 8.6% (an estimated of 99.9 million COPD patients).[16] On the other hand, with most areas located in mid-latitudes, China has experienced several severe cold spells in recent years. The cold spells in 2008 resulted in a significantly higher all-cause mortality in subtropical China and estimated losses exceeding $22.3 billion.[17, 18] Moreover, the public now has a better perception of the potential risks of extreme temperatures in China, especially those with chronic conditions.[19] Since no relevant studies have been reported in China, it is of great value to assess the association between cold spells and AECOPD hospitalizations to build prevention and adaption strategies suitable to local conditions (e.g., climate type, socio-demographic status of residents), which may be different from other regions.

Cold spells have been defined differently due to the heterogeneity of climate and

For peer review only

For peer review only - http://bmjopen.bmj.com/site/about/guidelines.xhtml

people's adaptive capacities in different regions. Previous studies suggested that the effects of cold spells varied by different cold spell characteristics and individual-specific factors.[20, 21] we have three main objectives in this work: (1) to illuminate the short-term effects of cold spells on the risk of hospitalizations for AECOPD with time-series methods; (2) to investigate the effect modification of cold spell intensities and durations by fitting different definitions and to explore the optimal cold spell definition in this region; (3) to identify potentially vulnerable populations through stratified analyses. The results could help better understand the relationship between extremely cold events and AECOPD hospitalizations, and provide scientific evidence in policymaking for local prevention and intervention of AECOPD.

MATERIALS AND METHODS

Data collection

Beijing, the capital of China, is located in the northern part of China (39°56′N, 116°20′E). The area covers 16410.54 km^2, with more than 21 million population in 2016. Beijing has a typical semi-humid continental monsoon climate with four distinctive seasons.

Daily hospitalizations for AECOPD from January 1, 2012, to December 31, 2016, were collected from the Beijing Public Health Information Center (http://www.phic.org.cn/). All government and private hospitals at the secondary or tertiary level in Beijing are required to submit their discharge records to the database.[22, 23] Each record consists of the following information: admission date, discharge date, age, gender, address, diagnosis and International Classification of Diseases 10th revision (ICD-10) diagnostic code. We excluded the records with missing or wrong information on residential addresses. Only those among Beijing residents admitted to hospitals with AECOPD as the primary

For peer review only - http://bmjopen.bmj.com/site/about/guidelines.xhtml

discharge diagnosis (ICD-10: J44) were included in the study.[23] The study was approved by the ethical committee of Peking Union Medical College Hospital. All the data for the analysis were anonymous at collection.

We collected the daily 2012–2016 meteorological data in Beijing from the China Meteorological Data Sharing Service System, including daily mean temperature (℃), daily mean relative humidity (%) and daily mean air pressure (hPa). For the same period, the daily air quality index (AQI) was obtained from the China National Environmental Monitoring Centre. The AQI value denotes the maximum value of individual air quality indexes (IAQI) of six monitored air pollutants (particulate matter with aerodynamic diameter<2.5μm ($PM_{2.5}$), particulate matter with aerodynamic diameter<10μm (PM_{10}), nitrogen dioxide, sulfur dioxide, carbon monoxide, and ozone). Considering the impact of influenza viral infectious on AECOPD,[13, 24] we also accessed the data of virological surveillance from the Chinese National Influenza Center (CNIC) (http://www.chinaivdc.cn/cnic/). The CNIC monitors the activity of seasonal influenza viruses in China and reports weekly positive rates of influenza isolations in the northern and southern parts separately. In this study, the onset of influenza epidemics (a binary variable representing days with relatively high influenza episodes)[25, 26] was defined as when the proportion of isolates positive for influenza in any given week exceeded 30% of the maximum weekly positive isolation rate in the whole surveillance season (Influenza surveillance season was defined from the 27th week of the previous year to the 26th week of the following year) in northern China.[27]

Cold spell definitions

The definition of cold spells varied across the research field due to distinct climatic features and temperature variations in different regions. As to the prior studies, cold

For peer review only

spells were usually defined based on their temperature thresholds and durations.[4, 15, 28] Instead of specific temperatures as thresholds, percentiles of temperature were shown to have a better model fit according to quasi-Poisson Akaike Information Criterion (Q-AIC).[6] Moreover, some researchers suggested that daily mean temperature is superior to the minimum or maximum temperature as an indicator to define cold spells because it reflects the exposure throughout the day rather than a short period.[20, 29] Therefore, we defined cold spell episodes as days when daily mean temperature at or below the 10th, 5th or 3rd percentile for at least 2, 3 or 4 consecutive days of the study period.[21] To avoid the possible biases caused by a few extreme summer events, we restricted the study period to the five coldest adjacent months (from November of the previous year to March of the following year) for each year.[6, 15, 20] The cold spell was treated as a dichotomous variable, with a value of 1 during the cold spell period. The statistical analyses were performed separately for each definition of cold spells.

Statistical methods

In the analyses, the dependent variable was the number of daily AECOPD hospitalizations following a quasi-Poisson distribution. Hence, we adopted a distributed lag model (DLM) [30] with a quasi-Poisson generalized linear regression model. To investigate the effects of cold spells on AECOPD hospitalizations, we compared the counts of AECOPD admissions during cold spell days with those during non-cold spell days, after adjusting for relative humidity, atmospheric pressure, AQI, seasonality, long-term trends, statutory holiday, influenza epidemics, and day of the week (DOW). The Q-AIC was employed to choose the optimal cold spell definition and degrees of freedom (df). The model was established as follows:

$$Y_t \sim \text{quasiPoisson}(\mu_t)$$

For peer review only - http://bmjopen.bmj.com/site/about/guidelines.xhtml

$$\text{Log}(\mu_t) = \alpha + \text{cb}(\text{CS}_t, \text{lag}, \text{df} = 3) + \text{ns}(\text{RH}_t, \text{df} = 3) + \text{ns}(\text{AP}_t, \text{df} = 3) + \text{ns}(\text{AQI}_t, \text{df} = 3)$$
$$+\text{ns}(\text{Time}_t, \text{df} = {}^{3}/\text{per year}) + \gamma\text{DOW}_t + \delta\text{Holiday}_t + \nu\text{Influenza}_t \; ,$$

where t is the day of observation; Y_t is the expected number of hospitalizations for AECOPD on day t; α is the intercept; CS_t denotes the cold spells on day t (0=non-cold spell days, and 1=cold spell days); cb represents the cross-basis function, including a linear function and a natural cubic spline function with 3 df to assess the linear and lagged effects of the cold spells separately. We fitted a lag structure up to 21 days in the models to completely capture the flexible lagged effects of cold spells exposure. ns refers to the natural cubic spline function. ns with 3 df was applied for the mean relative humidity (RH_t), mean atmospheric pressure (AP_t) and air quality index (AQI_t), respectively. ns with 3 df per year was used to control the seasonality and long-term trends. DOW_t is a categorical variable indicating the day of the week on day t, and γ is the coefficient. $Holiday_t$ is a binary variable (0=non-statutory holiday, and 1=statutory holiday) and δ is the coefficient. $Influenza_t$ is a dichotomous variable with the value of "1" for the influenza epidemic on day t, and ν is the coefficient. The statistical methods, maximum lag days and confounding factors included in the model were commonly utilized and described in previous publications.[4, 17, 20, 21, 31, 32]

To observe the variation trend of lagged effects, we calculated the single-day lagged effects (from lag0 to lag21) and cumulative effects (lag0, lag0–7, lag0–14 and lag0–21) of cold spells on AECOPD hospitalizations, respectively. To identify the susceptible subpopulations for more targeted public health interventions, we further conducted subgroup analyses to investigate the potential modification effects by gender (male and female) and age (0-64 years old and ≥65 years old) under the optimal definition of cold spells. The statistical differences of the risk estimates between the subgroups were examined by the Z-test with the following equation:

For peer review only

For peer review only - http://bmjopen.bmj.com/site/about/guidelines.xhtml

$$Z = (E_1 - E_2)/\sqrt{(SE_1{}^2 + SE_2{}^2)}$$

where Z represents the Z-test value; E_1 and E_2 denote the effect estimates of two categories; SE_1 and SE_2 are corresponding standard errors of E_1 and E_2.

Sensitivity analysis

We performed the sensitivity analyses by altering the df with 3–5 df per year of the long-term trend, 3–5 df of the relative humidity, 3-5 df of the air pressure, 3-5 df of the AQI and 3–5 df of the lag dimension in the DLM under the optimal definition of cold spells. We used R software version 3.6.1 with the "dlnm" and "splines" packages to run the analyses. All statistical tests were two-sided and values of $p<0.05$ were considered statistically significant.

Patient and public involvement

Patients were not involved in the development of the research question and outcome measures, study design or conduct of this study.

RESULTS

Data description

Table 1 shows the descriptive statistics of the study population, meteorological variables and AQI during the cold seasons (November to March) from 2012 to 2016 in Beijing. There was a total of 84,571 AECOPD hospitalizations throughout the study period. Among these cases, 63.6% were males and 36.4% were females. 83.9% of all patients were aged 65 years and above. The average of daily mean temperature, relative humidity, air pressure, and AQI were 0.9℃ (range, -16.0-18.0℃), 46.6% (range, 8.0-98.0%), 1025.3hPa (range, 1005.0-1044.0hPa) and 126.3 (range, 17.0-485.0),

For peer review only

For peer review only - http://bmjopen.bmj.com/site/about/guidelines.xhtml

respectively.

Table 2 shows the overview information of cold spells under different definitions. More days were defined as cold spell days with higher temperature thresholds and shorter duration. We observed the most cold spell episodes and days in four years with the definition of periods for at least 2 days and daily mean temperature below or at the 10th percentile (-6℃). In contrast, there were only 2 cold spell episodes (10 cold spell days) if we defined cold spells as periods for 4 or more consecutive days when daily mean temperature was below or at the 3rd percentile (-8℃).

Effects of cold spells under different definitions

Figure 1 depicts the lag structures of associations between cold spells under 9 different definitions and AECOPD hospitalizations of the total population. All cold spells had impacts on the risks of hospitalizations for AECOPD and most trends of their lagged effects were non-linear with two patterns. One was that the relative risk (RR) of hospitalizations for AECOPD reached a maximum on the days (lag0) of cold spells, then decreased and remained significant for 10-16 days (lag10-lag16). The other one was that the RR became significant on the 3rd or 4th day (lag3 or lag4) after exposure to cold spells, then gradually reached the maximum at about the 8th day (lag8) and lasted till the 15th-17th days (lag15-lag17).

Table 3 shows the cumulative effects of cold spells on AECOPD hospitalizations under different definitions. For each definition, the cumulative relative risk (CRR) increased with longer cumulative lags, with the highest CRR at lag0-21. Among 9 different definitions, the CRRs of cold spells at lag0-21 increased as the definition had a longer duration or lower temperature threshold. The maximum CRR over lag0-21 when the temperature threshold was set at the 10th percentile appeared at the duration≥4 consecutive days. In

For peer review only - http://bmjopen.bmj.com/site/about/guidelines.xhtml

comparison, the CRR values of the duration≥3 consecutive days and ≥2 consecutive days were lower. Likewise, when the duration was set for at least 4 consecutive days, the maximum CRR over lag0-21 appeared at the temperature range defined as ≤3rd percentile, while the CRRs of temperature threshold at the 5th and 10th percentile were lower. The lowest Q-AIC value (7769.8) indicating the best model fit was observed in Model 3 (Table 2). Hence, the optimal cold spell definition was daily mean temperature ≤10th percentile (-6℃) with at least 4 consecutive days during the study period. The optimal model was able to find the most significant single-day lagged effect earliest (lag0) and remained significant until the 14th day (lag14) (Figure 1).

Table 4 and Figure 2 reveal the results for the subgroup analyses of gender and age based on the optimal cold spell definition. The effects of cold spells were similar between males and females, with the most significant single-day lagged effect both occurred at lag0 (Z=0.041, P=0.48). The cumulative effects at lag0-21 of the two genders also differed slightly (Z=-0.730, P=0.23). Additionally, in the subgroups stratified by the age, the most significant single-day lagged effect and cumulative effect for people aged≥65 years were at lag0 and lag0-21, respectively. However, no significant effect of cold spells was observed in those aged 0-64 years. The results of sensitivity analyses indicated that the effect estimates of cold spells under the optimal definition on AECOPD hospitalizations were still robust. (See Supplementary Materials, Tables S1–S5).

DISCUSSION

In this study, we showed that cold spells were associated with increase hospitalizations for AECOPD. The adverse impacts of cold spells varied with their durations and intensities. Based on the statistical model fit, the optimal definition of cold spells was daily mean temperature less than or equal to the 10th percentile lasting for at least 4

For peer review only - http://bmjopen.bmj.com/site/about/guidelines.xhtml

consecutive days during the study period. The elderly seemed more sensitive to cold spells than the younger, while the susceptibility difference between genders was not noticeable.

Our finding is in accordance with previous studies reporting an excess of AECOPD hospitalizations,[15] emergency visits[33] and mortality[4, 6, 33] associated with cold spells. For instance, Monteiro et al. reported significant effects of cold spells identified by various indices on COPD hospitalizations with a lagged effect of at least two weeks.[15] Several underlying mechanisms may explain for elevated COPD morbidity and mortality attributable to extremely cold events. Firstly, cold exposure has been found to be related to a decline in lung function (FEV_1, FVC and PEF) among COPD patients.[34, 35] Secondly, Koskela et al. reported that cold air could directly induce bronchoconstriction, leading to excessive dyspnea in patients with COPD.[36] Thirdly, cold exposure may suppress the immune response and increase susceptibility to viral infections in humans.[37] Meanwhile, the transmission efficiency of the influenza virus is inversely correlated with ambient temperature.[38] Fourthly, the cold temperature may provoke airway inflammation and mucin hypersecretion in airway epithelium, which results in COPD morbidity and mortality by blocking airways and causing recurrent infections.[39, 40]

We found that the CRR values of AECOPD hospitalizations increased with longer duration and higher intensity of cold spells. Some prior studies had similar findings,[20, 21] indicating that both the duration and the intensity affect the health risks of cold spells. According to the minimum Q-AIC value criterion, the optimal definition of cold spells was the daily average temperature at or below the 10th percentile with 4 or more consecutive days. Compared with other definitions, this one had a lower intensity and longer duration, and the most significant single-day lagged effect of cold spells on AECOPD

For peer review only - http://bmjopen.bmj.com/site/about/guidelines.xhtml

hospitalizations appeared earliest (lag0). Our results agree with a study in Porto showing that the moderately low temperature with long periods contributed greater to COPD exacerbation than extremely low temperature with shorter-lasting days.[15] However, studies on some other diseases defined the optimal cold spell definitions with a threshold at the 5th percentile or at least 2 days duration,[21, 31] indicating different definitions may apply to different outcomes. In addition, some studies reported the temporal changes in people's adaption capacities to cold spells during recent decades under climate change.[5, 41] Overall, more effective cold spell definitions and warning systems adapted to regional climate, specific diseases, and dynamic changes of the population's sensitivity should be further studied and implemented in the future.

In the subgroup analyses based on the optimal cold spell definition, we found that the effects of cold spells on AECOPD hospitalizations were more significant in the elderly (aged ≥65 years) than people aged 0–64 years. This finding is consistent with previous studies.[4, 12, 42] The reasons may point to reduced thermoregulatory ability, higher prevalence of chronic diseases[20, 33] and impaired immunity[43] in the elderly. Note that since aging is one of the risk factors of COPD, most patients in our study were over 64, giving more power to achieve statistical significance. However, some studies showed the opposite results.[6] It was speculated that the younger tend to spend more time outdoors, which increase opportunities for exposure to extremely cold temperature. In terms of gender, we found similar impacts of cold spells among males and females, which corresponds with previous studies.[4, 12, 42]

Our study has significant public health implications for local prevention of AECOPD, development of early warning systems and rational allocation of medical and health resources to mitigate the COPD burden caused by cold spells. We showed substantial effects of cold spells on the risk of AECOPD hospitalizations with a lagged effect of about

For peer review only

For peer review only - http://bmjopen.bmj.com/site/about/guidelines.xhtml

3 weeks, urging for effective and practical guidelines for preventions, particularly for COPD patients during cold spells in China. Both the government and individuals should take practical actions. The meteorological departments should improve early warning systems with timely forecast and publication of extremely cold events. Moreover, the government should exert great efforts to raise public awareness of the health hazards of cold spells and ensure adequate public and medical services coping with cold spells.[4] As for individuals, it has been reported that staying indoor and wearing warm clothing could reduce mortality in extremely cold weather.[44] Tseng et al. suggested that COPD patients who received inhaled medicine were less affected by cold temperature-related COPD exacerbation.[12] Therefore, keeping warm, minimizing the outdoor activities and taking medications regularly are vital measures to fight against cold spells for individuals with COPD, especially for the elderly.

The main strengths of our study are as follows: Firstly, to the best of our knowledge, this is the first study to investigate the relationship between cold spells and AECOPD hospitalizations in China. Secondly, we controlled air quality and influenza epidemics as confounding factors, which were not included by some previous studies. Lee et al. have reported that a higher influenza virus detection rate was correlated with AECOPD.[14] A previous study from Beijing has found significant associations between short-term exposures to air pollution and hospitalizations for AECOPD.[23] Existing literatures have also shown that both air pollution and influenza epidemics could contribute to cold-related health effects.[45, 46] Moreover, air pollutants may interact with viral infections to precipitate AECOPD rather than acting alone. Feng and colleagues reported that ambient $PM_{2.5}$ was associated with influenza-like illness risk in Beijing in the flu season.[47] Further research on the interactions among cold spells, air pollution and influenza on AECOPD is therefore needed. Thirdly, we identified the elderly more vulnerable to cold

For peer review only - http://bmjopen.bmj.com/site/about/guidelines.xhtml

spells by stratified analyses, which guide more targeted prevention strategies.

Meanwhile, the study also has several limitations: Firstly, as an ecological study, the association between cold spells and AECOPD hospitalizations does not imply causality. Secondly, different socio-economic status or other factors on an individual level might be confounding factors and were not considered in the association. Thirdly, the meteorological variables and AQI were all from monitoring stations, not reflecting the individual level of exposures. Moreover, people are more likely to stay indoors with heating systems during extremely cold days in northern China, so inevitable exposure measurement errors may lead to bias. Fourthly, due to the limited availability of local data, the positive rates of influenza isolations were from the northern part of China but not only Beijing. Further studies with local influenza data included as a continuous variable in the model are warranted. Lastly, the data from only one city weakens the extrapolation validity of the study.

CONCLUSION

Our study demonstrates that short-term exposure to cold spells is associated with an increased risk of AECOPD hospitalizations. The cumulative effects increased with the intensities and the durations of cold spells. The elderly are more vulnerable to AECOPD hospitalizations during cold spell periods. These findings provide scientific foundations for comprehensive public health strategies to reduce cold spell-related AECOPD hospitalizations in Beijing, China.

Contributors: ZF obtained the original data and funding. ZF, XJ, YL and YC designed the study. DK, XL, JF, YZ and ZC preprocessed the data. YL, YC, and YZ analyzed the data. YL and YC drafted the manuscript. XZ, KX, CJ and ZF reviewed and edited the

For peer review only - http://bmjopen.bmj.com/site/about/guidelines.xhtml

manuscript. All authors have read and approved the final manuscript. ZF is the study guarantor.

Funding: This work was supported by National Key Research and Development Plan [2017YFC0211703], National 973 Project [2015CB553400], Chinese Academy of Medical Sciences (CAMS) Initiative for Innovative Medicine [CAMS 2016ZX310181-5/4, CAMS 2017-I2M-2-001], and National Natural Science Foundation [91643208, 41450006].

Competing interests: None declared.

Ethical approval: This study was approved by the ethical review committee of Peking Union Medical College Hospital.

Data sharing statement: No additional data are available.

For peer review only

For peer review only - http://bmjopen.bmj.com/site/about/guidelines.xhtml

REFERENCES

1 IPCC, 2014. *Climate Change 2014: Synthesis Report. Contribution of Working Groups I, II and III to the Fifth Assessment Report of the Intergovernmental Panel on Climate Change* [Core Writing Team, R.K. Pachauri and L.A. Meyer (eds.)]. IPCC, Geneva, Switzerland, 151 pp.

2 Cohen J, Screen JA, Furtado JC*, et al.* Recent Arctic amplification and extreme mid-latitude weather. *Nature Geoscience* 2014;**7**:627-37.

3 Zhang J, Tian W, Chipperfield MP*, et al.* Persistent shift of the Arctic polar vortex towards the Eurasian continent in recent decades. *Nature Climate Change* 2016;**6**:1094-9.

4 Chen J, Yang J, Zhou M*, et al.* Cold spell and mortality in 31 Chinese capital cities: Definitions, vulnerability and implications. *Environment international* 2019;**128**:271-8.

5 Lee W, Choi HM, Lee JY*, et al.* Temporal changes in mortality impacts of heat wave and cold spell in Korea and Japan. *Environment international* 2018;**116**:136-46.

6 Han J, Liu S, Zhang J*, et al.* The impact of temperature extremes on mortality: a time-series study in Jinan, China. *BMJ open* 2017;**7**:e014741.

7 Rabe KF, Watz H. Chronic obstructive pulmonary disease. *The Lancet* 2017;**389**:1931-40.

8 Lopez-Campos JL, Tan W, Soriano JB. Global burden of COPD. *Respirology* 2016;**21**:14-23.

9 Donaldson GC, Goldring JJ, Wedzicha JA. Influence of season on exacerbation characteristics in patients with COPD. *Chest* 2012;**141**:94-100.

10 McCormack MC, Paulin LM, Gummerson CE*, et al.* Colder temperature is associated with increased COPD morbidity. *Eur Respir J* 2017;**49**:1601501.

11 Chaturvedi S, Tseng C-M, Chen Y-T*, et al.* The Effect of Cold Temperature on Increased Exacerbation of Chronic Obstructive Pulmonary Disease: A Nationwide Study. *PloS one* 2013;**8**: e57066.

12 Tseng CM, Chen YT, Ou SM*, et al.* The effect of cold temperature on increased exacerbation of chronic obstructive pulmonary disease: a nationwide study. *PloS one* 2013;**8**:e57066.

13 Lee J, Jung HM, Kim SK, et al. Factors associated with chronic obstructive pulmonary disease exacerbation, based on big data analysis. Scientific reports 2019;**9**:6679.

14 Hajat S, Armstrong B, Baccini M*, et al.* Impact of high temperatures on mortality: is there an added heat wave effect? *Epidemiology (Cambridge, Mass)* 2006;**17**:632-8.

15 Monteiro A, Carvalho V, Gois J*, et al.* Use of "Cold Spell" indices to quantify excess chronic obstructive pulmonary disease (COPD) morbidity during winter (November to March 2000-2007): case study in Porto. *International journal of biometeorology* 2013;**57**:857-70.

16 Wang C, Xu J, Yang L*, et al.* Prevalence and risk factors of chronic obstructive pulmonary disease in China (the China Pulmonary Health [CPH] study): a national cross-sectional study. *The Lancet* 2018;**391**:1706-17.

For peer review only - http://bmjopen.bmj.com/site/about/guidelines.xhtml

17 Xie H, Yao Z, Zhang Y*, et al.* Short-term effects of the 2008 cold spell on mortality in three subtropical cities in Guangdong Province, China. *Environmental health perspectives* 2013;**121**:210-6.

18 Zhou B, Gu L, Ding Y*, et al.* The Great 2008 Chinese Ice Storm: Its Socioeconomic–Ecological Impact and Sustainability Lessons Learned. *Bulletin of the American Meteorological Society* 2011;**92**:47-60.

19 Ban J, Huang L, Chen C*, et al.* Integrating new indicators of predictors that shape the public's perception of local extreme temperature in China. *The Science of the total environment* 2017;**579**:529-36.

20 Wang L, Liu T, Hu M*, et al.* The impact of cold spells on mortality and effect modification by cold spell characteristics. *Scientific reports* 2016;**6**:38380.

21 Gao J, Yu F, Xu Z*, et al.* The association between cold spells and admissions of ischemic stroke in Hefei, China: Modified by gender and age. *The Science of the total environment* 2019;**669**:140-7.

22 Liu X, Kong D, Fu J*, et al.* Association between extreme temperature and acute myocardial infarction hospital admissions in Beijing, China: 2013-2016. *PloS one* 2018;**13**:e0204706.

23 Liang L, Cai Y, Barratt B*, et al.* Associations between daily air quality and hospitalisations for acute exacerbation of chronic obstructive pulmonary disease in Beijing, 2013–17: an ecological analysis. *The Lancet Planetary Health* 2019;**3**:e270-e9.

24 Wedzicha JA, Seemungal TAR. COPD exacerbations: defining their cause and prevention. *The Lancet* 2007;**370**:786-96.25 Solimini AG, Renzi M. Association between Air Pollution and Emergency Room Visits for Atrial Fibrillation. *International journal of environmental research and public health* 2017;**14**:661.

26 Stafoggia M, Samoli E, Alessandrini E*, et al.* Short-term associations between fine and coarse particulate matter and hospitalizations in Southern Europe: results from the MED-PARTICLES project. *Environmental health perspectives* 2013;**121**:1026-33.

27 Cowling BJ, Wong IO, Ho LM*, et al.* Methods for monitoring influenza surveillance data. *Int J Epidemiol* 2006;**35**:1314-21.

28 Song X, Wang S, Li T*, et al.* The impact of heat waves and cold spells on respiratory emergency department visits in Beijing, China. *The Science of the total environment* 2018;**615**:1499-505.

29 Guo Y, Jiang F, Peng L*, et al.* The association between cold spells and pediatric outpatient visits for asthma in Shanghai, China. *PloS one* 2012;**7**:e42232.

30 Gasparrini A. Modeling exposure-lag-response associations with distributed lag non-linear models. *Stat Med* 2014;**33**:881-99.

31 Cheng Q, Wang X, Wei Q*, et al.* The short-term effects of cold spells on pediatric outpatient admission for allergic rhinitis in Hefei, China. *The Science of the total environment* 2019;**664**:374-80.

For peer review only - http://bmjopen.bmj.com/site/about/guidelines.xhtml

32 Liang Z, Wang P, Zhao Q*, et al.* Effect of the 2008 cold spell on preterm births in two subtropical cities of Guangdong Province, Southern China. *The Science of the total environment* 2018;**642**:307-13.

33 de'Donato FK, Leone M, Noce D*, et al.* The impact of the February 2012 cold spell on health in Italy using surveillance data. *PloS one* 2013;**8**:e61720.

34 Lin Z, Gu Y, Liu C*, et al.* Effects of ambient temperature on lung function in patients with chronic obstructive pulmonary disease: A time-series panel study. *The Science of the total environment* 2018;**619-620**:360-5.

35 Donaldson GC, Seemungal T, Jeffries DJ*, et al.* Effect of temperature on lung function and symptoms in chronic obstructive pulmonary disease. *Eur Respir J* 1999;**13**:844-9.

36 Koskela HO, Koskela AK, Tukiaineu HO. Bronchoconstriction due to cold weather in COPD. The roles of direct airway effects and cutaneous reflex mechanisms. *Chest* 1996;**110**:632-6.

37 Shephard RJ, Shek PN. Cold exposure and immune function. *Can J Physiol Pharmacol* 1998;**76**:828-36.

38 Lowen AC, Mubareka S, Steel J*, et al.* Influenza virus transmission is dependent on relative humidity and temperature. *PLoS pathogens* 2007;**3**:1470-6.

39 Li M, Li Q, Yang G*, et al.* Cold temperature induces mucin hypersecretion from normal human bronchial epithelial cells in vitro through a transient receptor potential melastatin 8 (TRPM8)-mediated mechanism. *J Allergy Clin Immunol* 2011;**128**:626-34.e1-5.

40 Juan Y, Haiqiao W, Xie W*, et al.* Cold-inducible RNA-binding protein mediates airway inflammation and mucus hypersecretion through a post-transcriptional regulatory mechanism under cold stress. *Int J Biochem Cell Biol* 2016;**78**:335-48.

41 Chung Y, Noh H, Honda Y*, et al.* Temporal Changes in Mortality Related to Extreme Temperatures for 15 Cities in Northeast Asia: Adaptation to Heat and Maladaptation to Cold. *American journal of epidemiology* 2017;**185**:907-13.

42 Ma W, Yang C, Chu C*, et al.* The impact of the 2008 cold spell on mortality in Shanghai, China. *International journal of biometeorology* 2013;**57**:179-84.

43 Goodwin JS. Decreased immunity and increased morbidity in the elderly. *Nutr Rev* 1995;**53**:S41-4; discussion S4-6.

44 Donaldson GC, Ermakov SP, Komarov YM*, et al.* Cold related mortalities and protection against cold in Yakutsk, eastern Siberia: observation and interview study. *BMJ* 1998;**317**:978-82.

45 Qiu H, Tan K, Long F*, et al.* The Burden of COPD Morbidity Attributable to the Interaction between Ambient Air Pollution and Temperature in Chengdu, China. *International journal of environmental research and public health* 2018;**15**:492.

46 Huynen MM, Martens P, Schram D*, et al.* The impact of heat waves and cold spells on mortality rates in the Dutch population. *Environmental health perspectives* 2001;**109**:463-70.

47 Feng C, Li J, Sun W*, et al.* Impact of ambient fine particulate matter (PM2.5) exposure on

For peer review only - http://bmjopen.bmj.com/site/about/guidelines.xhtml

the risk of influenza-like-illness: a time-series analysis in Beijing, China. *Environmental health : a global access science source* 2016;**15**:17.

Figure legend/caption:

Figure 1 Lag-response relationships between cold spells under different definitions and AECOPD hospitalizations of the total population in Beijing, 2012-2016.

Figure 2 Lag-response relationships between cold spells and AECOPD hospitalizations stratified by gender and age in Beijing, 2012-2016.

For peer review only

For peer review only - http://bmjopen.bmj.com/site/about/guidelines.xhtml

Table 1 Statistic summary of daily hospitalizations for AECOPD (counts per day), meteorological variables and air quality index in cold seasons (November-March) in Beijing, China, 2012–2016.

	Total	Mean (SD)	Min	P25	Median	P75	Max
AECOPD hospitalizations	84571	111.7(41.0)	22.0	78.0	113.0	141.0	226.0
Gender							
Male	53829	71.1(25.8)	12.0	49.0	75.0	89.0	158.0
Female	30742	40.6(17.7)	7.0	27.0	39.0	53.0	110.0
Age							
0-64 years old	13600	18.0(8.3)	2.0	12.0	18.0	23.0	43.0
≥65 years old	70971	93.8(34.3)	19.0	67.0	95.0	118.0	189.0
Environmental variables							
Mean temperature (℃)	/	0.9(5.4)	-16.0	-3.0	0.0	4.0	18.0
Relative humidity (%)	/	46.6(20.4)	8.0	30.0	43.0	61.0	98.0
Air pressure (hPa)	/	1025.3(6.5)	1005.0	1021.0	1026.0	1030.0	1044.0
Air quality index	/	126.3(89.2)	17.0	61.0	97.0	170.0	485.0

AECOPD, acute exacerbation of chronic obstructive pulmonary disease; P25, the 25th percentile; P75, the 75th percentile; SD, standard deviation.

For peer review only - http://bmjopen.bmj.com/site/about/guidelines.xhtml

For peer review only

Table 2 Overview information of cold spells under different definitions

Model	Temperature threshold	Duration	Cold spell episodes	Cold spell days	Non-cold spell days	Q-AIC value
1	≤10% (-6℃)	≥2d	17	77	680	7819.6
2	≤10% (-6℃)	≥3d	11	65	692	7799.7
3	≤10% (-6℃)	≥4d	6	50	707	7769.8
4	≤5% (-7℃)	≥2d	8	37	720	7794.3
5	≤5% (-7℃)	≥3d	5	31	726	7786.6
6	≤5% (-7℃)	≥4d	3	25	732	7782.5
7	≤3% (-8℃)	≥2d	7	23	734	7786.5
8	≤3% (-8℃)	≥3d	5	19	738	7789.8
9	≤3% (-8℃)	≥4d	2	10	747	7804.4

Q-AIC, quasi-Poisson Akaike Information Criterion.

For peer review only

For peer review only - http://bmjopen.bmj.com/site/about/guidelines.xhtml

Table 3 Cumulative relative risk of cold spells under different definitions on AECOPD hospitalizations of the total population in Beijing, 2012-2016.

Definition	CRR (95% CI)			
	Lag0	Lag0-7	Lag0-14	Lag0-21
≤10% & ≥2d	1.030 (1.002-1.058)*	1.200 (1.087-1.326)*	1.278 (1.134-1.439)*	1.236 (1.055-1.448)*
≤10% & ≥3d	1.027 (1.000-1.054)*	1.188 (1.084-1.303)*	1.299 (1.166-1.448)*	1.353 (1.161-1.577)*
≤10% &≥4d	1.042 (1.013-1.072)*	1.249 (1.136-1.374)*	1.343 (1.206-1.496)*	1.394 (1.193-1.630)*
≤5% & ≥2d	1.012 (0.980-1.045)	1.173 (1.055-1.304)*	1.344 (1.188-1.519)*	1.354 (1.141-1.608)*
≤5% & ≥3d	1.031 (1.000-1.063)*	1.235 (1.114-1.369)*	1.381 (1.220-1.563)*	1.428 (1.206-1.692)*
≤5% & ≥4d	1.042 (1.006-1.079)*	1.268 (1.132-1.421)*	1.404 (1.234-1.598)*	1.511 (1.262-1.809)*
≤3% & ≥2d	0.982 (0.944-1.021)	1.113 (0.960-1.290)	1.412 (1.171-1.703)*	1.444 (1.113-1.873)*
≤3% & ≥3d	1.009 (0.970-1.049)	1.217 (1.051-1.411)*	1.525 (1.264-1.841)*	1.659 (1.278-2.154)*
≤3% & ≥4d	0.992 (0.951-1.035)	1.201 (0.999-1.443)	1.644 (1.261-2.145)*	1.889 (1.315-2.712)*

AECOPD, acute exacerbation of chronic obstructive pulmonary disease; CI, confidence interval; CRR, cumulative relative risk.

*p<0.05.

For peer review only - http://bmjopen.bmj.com/site/about/guidelines.xhtml

Table 4 Cumulative relative risk of cold spells# on AECOPD hospitalizations stratified by gender and age in Beijing, 2012-2016.

Subgroup	CRR (95% CI)			
	Lag0	Lag0-7	Lag0-14	Lag0-21
Male	1.042 (1.011-1.074)*	1.243 (1.123-1.375)*	1.316 (1.173-1.477)*	1.342 (1.136-1.586)*
Female	1.041 (1.005-1.077)*	1.257 (1.119-1.411)*	1.383 (1.215-1.574)*	1.476 (1.211-1.783)*
Age<65	1.017 (0.972-1.064)	1.120 (0.963-1.303)	1.159 (0.977-1.376)	1.107 (0.862-1.422)
Age≥65	1.046 (1.017-1.077)*	1.275 (1.158-1.404)*	1.382 (1.240-1.540)*	1.456 (1.244-1.705)*

AECOPD, acute exacerbation of chronic obstructive pulmonary disease; CI, confidence interval; CRR, cumulative relative risk.

#The optimal cold spell definition was daily mean temperature ≤10th percentile (-6℃) with at least 4 consecutive days during the study period.

*p<0.05.

For peer review only

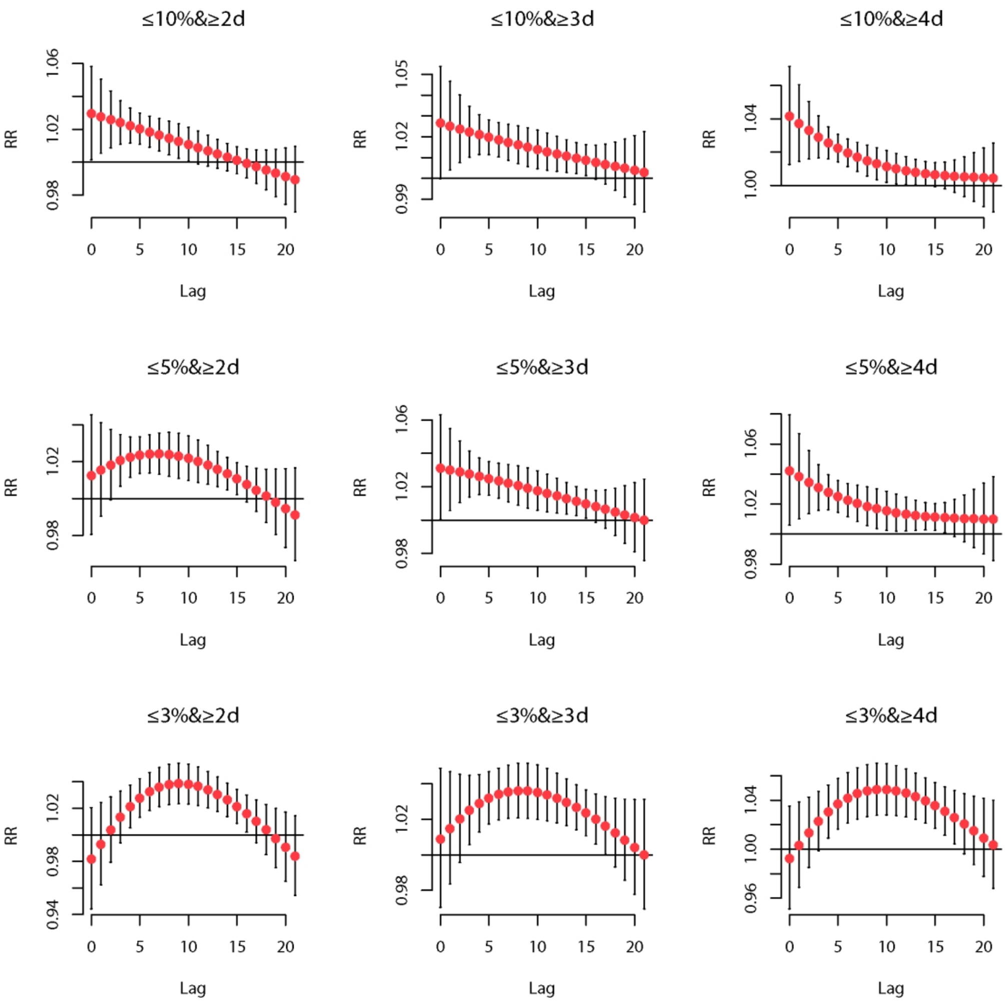

Figure 1 Lag-response relationships between cold spells under different definitions and AECOPD hospitalizations of the total population in Beijing, 2012-2016.

161x159mm (300 x 300 DPI)

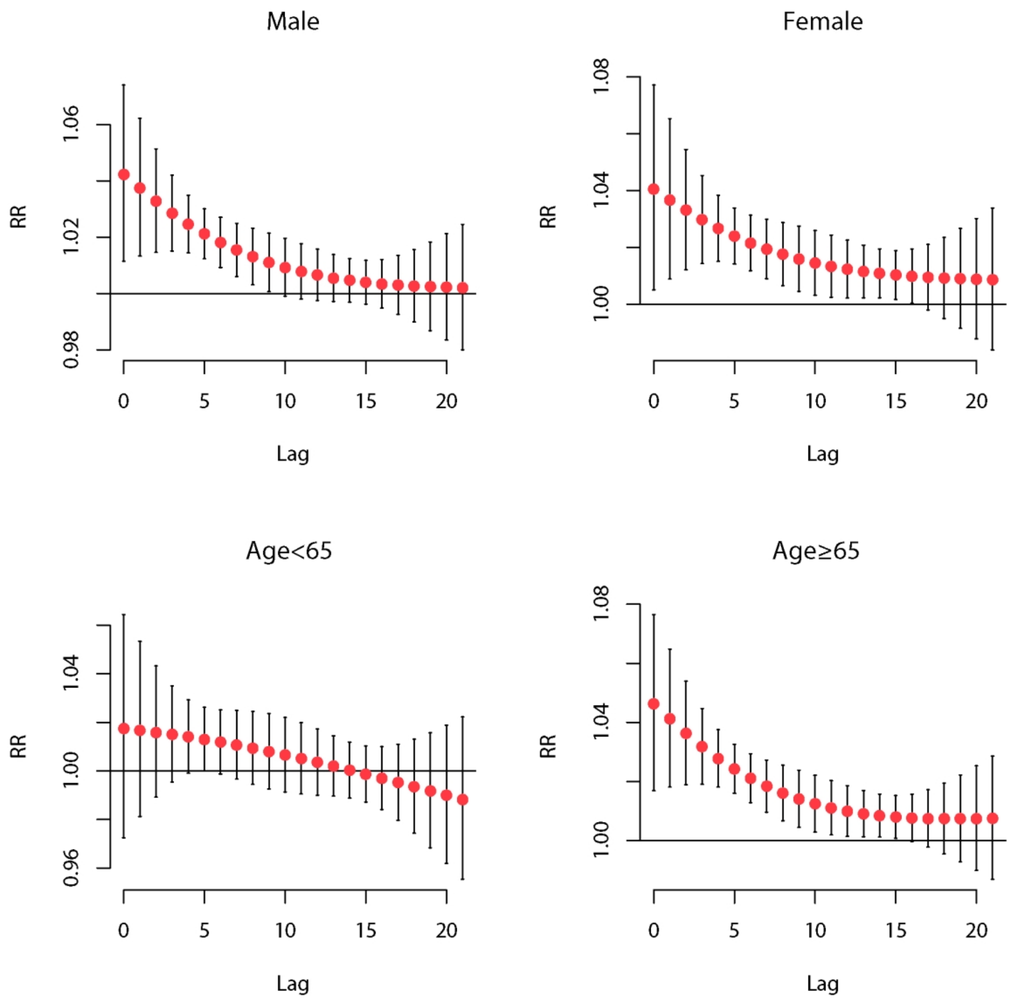

Figure 2 Lag-response relationships between cold spells and AECOPD hospitalizations stratified by gender and age in Beijing, 2012-2016.

159x157mm (300 x 300 DPI)

Supplementary Materials

Supplementary Tables

Table S1 The cumulative effects of cold spells under the optimal definition using different degrees of freedom for lag dimension in the DLM model

Table S2 The cumulative effects of cold spells under the optimal definition using different degrees of freedom for time per year in the DLM model

Table S3 The cumulative effects of cold spells under the optimal definition using different degrees of freedom for relative humidity in the DLM model

Table S4 The cumulative effects of cold spells under the optimal definition using different degrees of freedom for air pressure in the DLM model

Table S5 The cumulative effects of cold spells under the optimal definition using different degrees of freedom for air quality index in the DLM model

For peer review only

Table S1 The cumulative effects of cold spells under the optimal definition using different degrees of freedom for lag dimension in the DLM model

df for lag dimension	Group	CRR (95% CI)			
		Lag0	Lag0-7	Lag0-14	Lag0-21
3[a]	Total	1.042 (1.013-1.072)*	1.249 (1.136-1.374)*	1.343 (1.206-1.496)*	1.394 (1.193-1.630)*
	Male	1.042 (1.011-1.074)*	1.243 (1.123-1.375)*	1.316 (1.173-1.477)*	1.342 (1.136-1.586)*
	Female	1.041 (1.005-1.077)*	1.257 (1.119-1.411)*	1.383 (1.215-1.574)*	1.476 (1.211-1.783)*
	Age<65	1.017 (0.972-1.064)	1.120 (0.963-1.303)	1.159 (0.977-1.376)	1.107 (0.862-1.422)
	Age⩾65	1.046 (1.017-1.077)*	1.275 (1.158-1.404)*	1.382 (1.240-1.540)*	1.456 (1.244-1.705)*
4	Total	0.989 (0.926-1.057)	1.265 (1.149-1.392)*	1.311 (1.173-1.465)*	1.382 (1.182-1.615)*
	Male	0.992 (0.925-1.064)	1.257 (1.135-1.393)*	1.285 (1.141-1.448)*	1.331 (1.126-1.573)*
	Female	0.983 (0.906-1.067)	1.274 (1.133-1.432)*	1.347 (1.178-1.540)*	1.462 (1.210-1.766)*
	Age<65	0.919 (0.827-1.022)	1.151 (0.988-1.340)	1.106 (0.926-1.320)	1.091 (0.850-1.401)
	Age⩾65	1.003 (0.938-1.073)	1.288 (1.169-1.419)*	1.355 (1.211-1.515)*	1.446 (1.235-1.692)*
5	Total	1.001 (0.888-1.128)	1.271 (1.150-1.404)*	1.313 (1.174-1.468)*	1.389 (1.185-1.628)*
	Male	0.999 (0.880-1.136)	1.261 (1.134-1.403)*	1.287 (1.141-1.451)*	1.336 (1.127-1.583)*
	Female	1.004 (0.868-1.161)	1.285 (1.137-1.451)*	1.350 (1.180-1.545)*	1.474 (1.216-1.787)*
	Age<65	0.892 (0.737-1.080)	1.143 (0.975-1.341)	1.102 (0.922-1.317)	1.084 (0.840-1.398)
	Age⩾65	1.023 (0.907-1.155)	1.297 (1.172-1.434)*	1.358 (1.213-1.520)*	1.456 (1.240-1.710)*

CI, confidence interval; df, degree of freedom; RR, relative risk.

*P<0.05.

[a]Used in the study.

For peer review only

Table S2 The cumulative effects of cold spells under the optimal definition using different degrees of freedom for time per year in the DLM model

df for time per year	Group	CRR (95% CI)			
		Lag0	Lag0-7	Lag0-14	Lag0-21
3[a]	Total	1.042 (1.013-1.072)*	1.249 (1.136-1.374)*	1.343 (1.206-1.496)*	1.394 (1.193-1.630)*
	Male	1.042 (1.011-1.074)*	1.243 (1.123-1.375)*	1.316 (1.173-1.477)*	1.342 (1.136-1.586)*
	Female	1.041 (1.005-1.077)*	1.257 (1.119-1.411)*	1.383 (1.215-1.574)*	1.476 (1.211-1.783)*
	Age<65	1.017 (0.972-1.064)	1.120 (0.963-1.303)	1.159 (0.977-1.376)	1.107 (0.862-1.422)
	Age⩾65	1.046 (1.017-1.077)*	1.275 (1.158-1.404)*	1.382 (1.240-1.540)*	1.456 (1.244-1.705)*
4	Total	1.023 (0.992-1.055)	1.150 (1.033-1.280)*	1.218 (1.078-1.376)*	1.233 (1.036-1.467)*
	Male	1.029 (0.997-1.063)	1.173 (1.045-1.316)*	1.226 (1.074-1.399)*	1.225 (1.015-1.480)*
	Female	1.011 (0.974-1.050)	1.107 (0.971-1.262)	1.196 (1.034-1.385)*	1.232 (0.998-1.520)
	Age<65	1.010 (0.961-1.061)	1.077 (0.906-1.280)	1.105 (0.907-1.345)	1.056 (0.795-1.401)
	Age⩾65	1.026 (0.995-1.058)	1.166 (1.046-1.300)*	1.244 (1.100-1.406)*	1.272 (1.067-1.517)*
5	Total	1.022 (0.990-1.056)	1.144 (1.008-1.298)*	1.208 (1.031-1.415)*	1.222 (0.978-1.526)
	Male	1.030 (0.994-1.066)	1.171 (1.021-1.343)*	1.222 (1.027-1.452)*	1.223 (0.960-1.559)
	Female	1.009 (0.970-1.050)	1.093 (0.938-1.275)	1.174 (0.972-1.418)	1.203 (0.923-1.569)
	Age<65	1.011 (0.959-1.066)	1.082 (0.883-1.327)	1.127 (0.874-1.453)	1.127 (0.788-1.612)
	Age⩾65	1.025 (0.992-1.059)	1.158 (1.019-1.316)*	1.227 (1.045-1.440)*	1.245 (0.994-1.559)

CI, confidence interval; df, degree of freedom; RR, relative risk.
*P<0.05.
[a]Used in the study.

For peer review only

Table S3 The cumulative effects of cold spells under the optimal definition using different degrees of freedom for relative humidity in the DLM model

df for relative humidity	Group	CRR (95% CI)			
		Lag0	Lag0-7	Lag0-14	Lag0-21
3[a]	Total	1.042 (1.013-1.072)*	1.249 (1.136-1.374)*	1.343 (1.206-1.496)*	1.394 (1.193-1.630)*
	Male	1.042 (1.011-1.074)*	1.243 (1.123-1.375)*	1.316 (1.173-1.477)*	1.342 (1.136-1.586)*
	Female	1.041 (1.005-1.077)*	1.257 (1.119-1.411)*	1.383 (1.215-1.574)*	1.476 (1.211-1.783)*
	Age<65	1.017 (0.972-1.064)	1.120 (0.963-1.303)	1.159 (0.977-1.376)	1.107 (0.862-1.422)
	Age≥65	1.046 (1.017-1.077)*	1.275 (1.158-1.404)*	1.382 (1.240-1.540)*	1.456 (1.244-1.705)*
4	Total	1.041 (1.012-1.071)*	1.248 (1.135-1.373)*	1.343 (1.205-1.495)*	1.392 (1.190-1.629)*
	Male	1.042 (1.011-1.073)*	1.240 (1.120-1.372)*	1.314 (1.170-1.475)*	1.336 (1.130-1.580)*
	Female	1.041 (1.006-1.078)*	1.259 (1.120-1.414)*	1.385 (1.217-1.578)*	1.482 (1.225-1.792)*
	Age<65	1.017 (0.972-1.064)	1.119 (0.961-1.302)	1.158 (0.975-1.375)	1.105 (0.859-1.420)
	Age≥65	1.046 (1.017-1.076)*	1.274 (1.157-1.403)*	1.381 (1.239-1.540)*	1.455 (1.242-1.704)*
5	Total	1.041 (1.012-1.071)*	1.244 (1.131-1.369)*	1.337 (1.200-1.490)*	1.383 (1.182-1.619)*
	Male	1.041 (1.010-1.073)*	1.236 (1.116-1.368)*	1.307 (1.164-1.468)*	1.327 (1.121-1.569)*
	Female	1.040 (1.005-1.077)*	1.256 (1.117-1.411)*	1.381 (1.212-1.574)*	1.474 (1.218-1.785)*
	Age<65	1.015 (0.970-1.062)	1.111 (0.955-1.293)	1.148 (0.967-1.363)	1.089 (0.847-1.400)
	Age≥65	1.046 (1.016-1.076)*	1.271 (1.154-1.400)*	1.377 (1.234-1.536)*	1.448 (1.235-1.697)*

CI, confidence interval; df, degree of freedom; RR, relative risk.

*P<0.05.

[a]Used in the study.

For peer review only

Table S4 The cumulative effects of cold spells under the optimal definition using different degrees of freedom for air pressure in the DLM model

df for air pressure	Group	CRR (95% CI)			
		Lag0	Lag0-7	Lag0-14	Lag0-21
3[a]	Total	1.042 (1.013-1.072)*	1.249 (1.136-1.374)*	1.343 (1.206-1.496)*	1.394 (1.193-1.630)*
	Male	1.042 (1.011-1.074)*	1.243 (1.123-1.375)*	1.316 (1.173-1.477)*	1.342 (1.136-1.586)*
	Female	1.041 (1.005-1.077)*	1.257 (1.119-1.411)*	1.383 (1.215-1.574)*	1.476 (1.211-1.783)*
	Age<65	1.017 (0.972-1.064)	1.120 (0.963-1.303)	1.159 (0.977-1.376)	1.107 (0.862-1.422)
	Age≥65	1.046 (1.017-1.077)*	1.275 (1.158-1.404)*	1.382 (1.240-1.540)*	1.456 (1.244-1.705)*
4	Total	1.042 (1.012-1.071)*	1.248 (1.134-1.373)*	1.342 (1.205-1.495)*	1.393 (1.192-1.629)*
	Male	1.042 (1.011-1.074)*	1.240 (1.120-1.373)*	1.313 (1.170-1.475)*	1.340 (1.134-1.584)*
	Female	1.041 (1.005-1.078)*	1.259 (1.120-1.414)*	1.385 (1.216-1.578)*	1.478 (1.223-1.786)*
	Age<65	1.018 (0.973-1.065)	1.123 (0.965-1.306)	1.162 (0.978-1.380)	1.109 (0.863-1.425)
	Age≥65	1.046 (1.016-1.076)*	1.273 (1.156-1.402)*	1.380 (1.238-1.539)*	1.455 (1.242-1.704)*
5	Total	1.041 (1.012-1.071)*	1.247 (1.133-1.373)*	1.342 (1.204-1.495)*	1.393 (1.191-1.629)*
	Male	1.042 (1.011-1.074)*	1.240 (1.120-1.374)*	1.314 (1.170-1.476)*	1.341 (1.134-1.585)*
	Female	1.041 (1.005-1.077)*	1.257 (1.118-1.413)*	1.383 (1.214-1.576)*	1.475 (1.220-1.784)*
	Age<65	1.019 (0.974-1.067)	1.129 (0.970-1.314)	1.168 (0.984-1.388)	1.115 (0.868-1.433)
	Age≥65	1.046 (1.016-1.076)*	1.271 (1.154-1.401)*	1.378 (1.235-1.537)*	1.452 (1.240-1.701)*

CI, confidence interval; df, degree of freedom; RR, relative risk.

*P<0.05.

[a]Used in the study.

For peer review only

Table S5 The cumulative effects of cold spells under the optimal definition using different degrees of freedom for air quality index in the DLM model

df for AQI	Group	CRR (95% CI)			
		Lag0	Lag0-7	Lag0-14	Lag0-21
3[a]	Total	1.042 (1.013-1.072)*	1.249 (1.136-1.374)*	1.343 (1.206-1.496)*	1.394 (1.193-1.630)*
	Male	1.042 (1.011-1.074)*	1.243 (1.123-1.375)*	1.316 (1.173-1.477)*	1.342 (1.136-1.586)*
	Female	1.041 (1.005-1.077)*	1.257 (1.119-1.411)*	1.383 (1.215-1.574)*	1.476 (1.211-1.783)*
	Age<65	1.017 (0.972-1.064)	1.120 (0.963-1.303)	1.159 (0.977-1.376)	1.107 (0.862-1.422)
	Age≥65	1.046 (1.017-1.077)*	1.275 (1.158-1.404)*	1.382 (1.240-1.540)*	1.456 (1.244-1.705)*
4	Total	1.042 (1.013-1.072)*	1.249 (1.135-1.374)*	1.342 (1.205-1.495)*	1.393 (1.192-1.629)*
	Male	1.042 (1.012-1.074)*	1.242 (1.123-1.375)*	1.315 (1.171-1.476)*	1.342 (1.135-1.586)*
	Female	1.041 (1.005-1.077)*	1.257 (1.119-1.412)*	1.382 (1.214-1.573)*	1.475 (1.221-1.783)*
	Age<65	1.018 (0.973-1.065)	1.120 (0.963-1.303)	1.157 (0.974-1.373)	1.106 (0.861-1.421)
	Age≥65	1.046 (1.017-1.077)*	1.275 (1.158-1.404)*	1.381 (1.239-1.539)*	1.456 (1.243-1.704)*
5	Total	1.042 (1.013-1.071)*	1.249 (1.136-1.373)*	1.347 (1.210-1.500)*	1.405 (1.202-1.643)*
	Male	1.042 (1.011-1.074)*	1.242 (1.123-1.373)*	1.320 (1.177-1.480)*	1.355 (1.148-1.601)*
	Female	1.041 (1.005-1.077)*	1.257 (1.119-1.412)*	1.385 (1.216-1.577)*	1.482 (1.226-1.792)*
	Age<65	1.018 (0.973-1.065)	1.120 (0.963-1.303)	1.161 (0.978-1.378)	1.116 (0.869-1.433)
	Age≥65	1.046 (1.017-1.076)*	1.275 (1.159-1.403)*	1.386 (1.243-1.545)*	1.468 (1.254-1.718)*

AQI, air quality index; CI, confidence interval; df, degree of freedom; RR, relative risk.

*P<0.05.

[a]Used in the study.

For peer review only

STROBE Statement—Checklist of items that should be included in reports of ***cross-sectional studies***

	Item No	Recommendation	Page No.
**Title and abstract**	1	(*a*) Indicate the study's design with a commonly used term in the title or the abstract	1-2
		(*b*) Provide in the abstract an informative and balanced summary of what was done and what was found	2
**Introduction**			
Background/rationale	2	Explain the scientific background and rationale for the investigation being reported	3-4
Objectives	3	State specific objectives, including any prespecified hypotheses	4
**Methods**			
Study design	4	Present key elements of study design early in the paper	4-5
Setting	5	Describe the setting, locations, and relevant dates, including periods of recruitment, exposure, follow-up, and data collection	4-5
Participants	6	(*a*) Give the eligibility criteria, and the sources and methods of selection of participants	4
Variables	7	Clearly define all outcomes, exposures, predictors, potential confounders, and effect modifiers. Give diagnostic criteria, if applicable	4-6
Data sources/ measurement	8*	For each variable of interest, give sources of data and details of methods of assessment (measurement). Describe comparability of assessment methods if there is more than one group	4-6
Bias	9	Describe any efforts to address potential sources of bias	5
Study size	10	Explain how the study size was arrived at	NA
Quantitative variables	11	Explain how quantitative variables were handled in the analyses. If applicable, describe which groupings were chosen and why	NA
Statistical methods	12	(*a*) Describe all statistical methods, including those used to control for confounding	6-7
		(*b*) Describe any methods used to examine subgroups and interactions	7
		(*c*) Explain how missing data were addressed	4
		(*d*) If applicable, describe analytical methods taking account of sampling strategy	NA
		€ Describe any sensitivity analyses	7
**Results**			
Participants	13*	(a) Report numbers of individuals at each stage of study—eg numbers potentially eligible, examined for eligibility, confirmed eligible, included in the study, completing follow-up, and analysed	7
		(b) Give reasons for non-participation at each stage	NA
		(c) Consider use of a flow diagram	NA
Descriptive data	14*	(a) Give characteristics of study participants (eg demographic, clinical, social) and information on exposures and potential confounders	7-8
		(b) Indicate number of participants with missing data for each variable of interest	NA
Outcome data	15*	Report numbers of outcome events or summary measures	7
Main results	16	(*a*) Give unadjusted estimates and, if applicable, confounder-adjusted estimates and their precision (eg, 95% confidence interval). Make clear which confounders were adjusted for and why they were included	8-9

For peer review only

For peer review only - http://bmjopen.bmj.com/site/about/guidelines.xhtml

		(*b*) Report category boundaries when continuous variables were categorized	NA
		(*c*) If relevant, consider translating estimates of relative risk into absolute risk for a meaningful time period	NA
Other analyses	17	Report other analyses done—eg analyses of subgroups and interactions, and sensitivity analyses	9
**Discussion**			
Key results	18	Summarise key results with reference to study objectives	9
Limitations	19	Discuss limitations of the study, taking into account sources of potential bias or imprecision. Discuss both direction and magnitude of any potential bias	11-12
Interpretation	20	Give a cautious overall interpretation of results considering objectives, limitations, multiplicity of analyses, results from similar studies, and other relevant evidence	9-10
Generalisability	21	Discuss the generalisability (external validity) of the study results	12
**Other information**			
Funding	22	Give the source of funding and the role of the funders for the present study and, if applicable, for the original study on which the present article is based	12

*Give information separately for exposed and unexposed groups.

Note: An Explanation and Elaboration article discusses each checklist item and gives methodological background and published examples of transparent reporting. The STROBE checklist is best used in conjunction with this article (freely available on the Web sites of PLoS Medicine at http://www.plosmedicine.org/, Annals of Internal Medicine at http://www.annals.org/, and Epidemiology at http://www.epidem.com/). Information on the STROBE Initiative is available at www.strobe-statement.org.

For peer review only
